# The change in the pharmacological significance of dihydralazine (Germany) and hydralazine (USA)

**DOI:** 10.1007/s00210-025-04370-x

**Published:** 2025-07-09

**Authors:** Carina Mech, Roland Seifert

**Affiliations:** Institute of Pharmacology Hannover Medical School, Carl-Neuberg-Str. 1, 30625 Hannover, Germany

**Keywords:** Dihydralazine, Hydralazine, Textbook analysis, Rote Liste (Red List), Expert information, Guidelines

## Abstract

Dihydralazine and hydralazine have been on the market since the 1950s. The primary indication for those is the treatment of hypertension. The aim of this study is to uncover the reasons why both, dihydralazine in Germany and hydralazine in the USA, have lost importance. The period under investigation covers the years 1955 to 2025. The standard work from the USA *The Pharmacological Basis of Therapeutics* (Goodman & Gilman) and three German pharmacology textbooks were selected: *Allgemeine und spezielle Pharmakologie und Toxikologie* (Aktories), *Pharmakologie und Toxikologie* (Lüllmann) and *Allgemeine und Spezielle Pharmakologie und Toxikologie* (Karow). Categories were created for the German and US textbooks. Clinical studies were used to evaluate the textbooks. In addition, the “Rote Liste” (Red List) of drugs was examined for each decade to identify changes. Guidelines and specialist information were also analyzed. Furthermore, learning platforms were examined about the learning content on dihydralazine. The textbooks show that dihydralazine is now only a reserve drug. Hydralazine is also no longer the drug of choice in the USA. The reason for the change in the evaluation of (di)hydralazines is the large number of adverse drug reactions (ADRs). In Goodman & Gilman, the areas of application increased from 1955 to 2023, whereas German pharmacology textbooks (Aktories) and (Lüllmann) show a decrease in indications over time. The (di)hydralazines now have an indication for chronic heart failure (CHF) in African Americans. The dose of hydralazine in the US and dihydralazine in Germany has been reduced over time. The (di)hydralazines have a wide spectrum of ADRs. Dihydralazine is no longer on the German drug list for medical exams. However, it is still covered on learning platforms. Dihydralazine in Germany and hydralazine in the USA have lost pharmacological importance but they are still used in individual situations, if tolerated, to reduce blood presssure.

**Supplementary Information:**

The online version contains supplementary material available at 10.1007/s00210-025-04370-x.

## Introduction

Dihydralazine and hydralazine ((di)hydralazines) belong to the group of *antihypertensives* (Aktories et al. 2022). (Di)hydralazines are vasodilators (Aktories et al. 2022). Chemically speaking, (di)hydralazines are *phthalazines*. Both have a similar structure (10.1016/B978-0-444-52072-2.50012-4). Hydralazine has one hydrazine group, while dihydralazine has two hydrazine groups. The effects of hydralazine and dihydralazine are very similar (Morand et al. 1979). Dihydralazine has a longer half-life and lower oral bioavailability than hydralazine (Embryotox, https://www.embryotox.de/arzneimittel/details/ansicht/medikament/dihydralazin, accessed 06.05.2025). The drug class of vasodilators was chosen, because they have high potential to greatly reduce blood pressure. (Di)hydralazines were specifically selected, because they have been on the market for a long time (1950s). Hypertension is a widespread disease (Hochdruckliga, https://www.hochdruckliga.de/pressemitteilung/die-volkskrankheit-hypertonie-geht-alle-an, accessed 06.05.2025). It is therefore crucial for doctors and medical students to consider the historic development of the evaluation of drugs.

For the treatment of hypertension, hydralazine can be combined with a β-adrenoreceptor antagonist and a Na–K-2Cl cotransporter (NKCC) or NaCl cotransporter (NCC) inhibitor (Aktories et al. 2022). Nowadays, low doses of hydralazine are used (25–50 mg/day) (mg/day) (Aktories et al. 2022). Hydralazine was developed in the 1950 s for the treatment of malaria (PubChem, Hydralazine, https://pubchem.ncbi.nlm.nih.gov/compound/3637, accessed 22.04.2025). However, due to its antihypertensive effect, hydralazine was used as an antihypertensive drug (PubChem, Hydralazine, https://pubchem.ncbi.nlm.nih.gov/compound/3637, accessed 22.04.2025). Although (di)hydralazines have been around for decades, the mechanism of action has not yet been fully researched (Aktories et al. 2022). Dihydralazine is an effective drug (Wacker et al. 2006). Over time, however, drugs have come onto the market that are not only effective but also have fewer adverse drug reactions (ADRs) (Wacker et al. 2006). The large number of ADRs associated with hydralazine was investigated as early as 1973 (Perry 1973). Prolonged use of hydralazine can lead to Lupus Erythematosus (LE) (Perry 1973). Since 1996, dihydralazine has only been referred to as a *reserve drug* (Forth et al. 1996). For a long time, dihydralazine was the drug of choice for the treatment of pre-eclampsia (Bolte et al. 2001). Hydralazine was used for the treatment of heart failure with reduced ejection fraction in combination with isosorbide dinitrate (ISDN) (NIH, Hydralazine, https://www.ncbi.nlm.nih.gov/books/NBK470296/, accessed 22.04.2025). Dihydralazine combined with nitrates has an indication in African Americans for the treatment of heart failure (HF) (Aktories et al. 2022).

The aim of this paper is to investigate the reasons for the change in pharmacological importance of hydralazine and dihydralazine and to uncover differences between the USA and Germany.

## Material and methods

### Selection and analysis of pharmacology textbooks

**Fig. 1:**
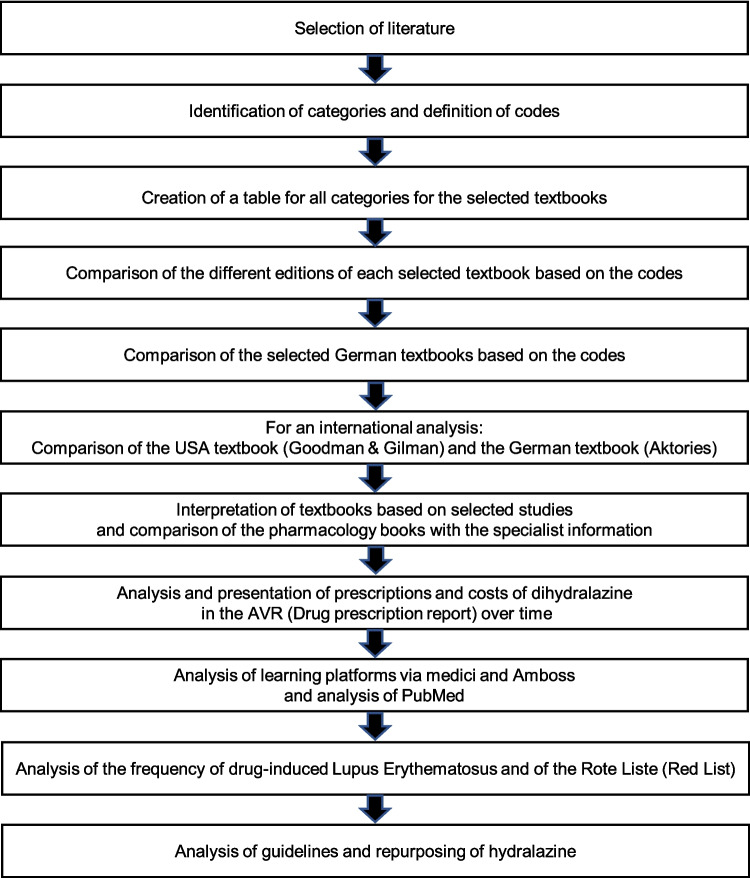
Illustration of the methodological approach

Various literature has been selected to investigate the relevance of dihydralazine and hydralazine (Fig. 1). Table S1 provides an overview of the textbook series used (Forth et al. 1975, 1977, 1980, 1983, 1987, 1992, 1996, 2001; Aktories et al. 2005, 2009, 2013, 2017, 2022; Kuschinsky and Lüllmann 1964, 1966, 1967, 1970, 1972, 1974, 1976, 1978, 1981, 1984, 1987, 1989,1993; Lüllmann and Mohr 1999; Lüllmann et al. 2003, 2006, 2010, 2016; Karow and Lang 1994, 1995, 1996, 1997, 1998, 1999, 2001, 2002; Karow and Lang-Roth 2003, 2004, 2005, 2006, 2007, 2008, 2009, 2010, 2011, 2012, 2013, 2014, 2015, 2016, 2017, 2018, 2019, 2020, 2021, 2022, 2023, 2024; Goodman and Gilman 1941, 1955, 1965, 1970, 1975, 1980; Goodman et al. 1985; Gilman et al. 1990; Hardman et al. 1996, 2001; Brunton et al. 2006, 2011, 2018; Brunton and Knollmann 2023). One selection criterion for the textbooks was that they had to have different editions. In addition, the editions of the books had to cover a period of at least 30 years to analyze the change in the significance of dihydralazine and hydralazine. Therefore, the following textbooks were selected: *Allgemeine und spezielle Pharmakologie und Toxikologie* (Aktories), *Pharmakologie und Toxikologie* (Lüllmann) and *Allgemeine und Spezielle Pharmakologie und Toxikologie* (Karow) as well as the standard work from the USA *The Pharmacological Basis of Therapeutics* (Goodman & Gilman). As Goodman & Gilman is considered as a standard work, only one textbook from the USA was selected.

The Goodman & Gilman textbook has 14 editions. The first edition was published in 1941 (Goodman and Gilman 1941). Hydralazine is not yet mentioned in this edition. Hydralazine is not mentioned until the second edition (1955) (Goodman and Gilman 1955). The 14th edition was published in 2023 (Brunton and Knollmann 2023). Each edition of the textbook was available for analysis. The German textbook *Allgemeine und spezielle Pharmakologie und Toxikologie* (Aktories) has 13 editions. The first edition was published in 1975 (Forth et al. 1975) The last edition was published in 2022 (Aktories et al. 2022). The first edition of the textbook *Pharmacology and Toxicology* (Lüllmann) was published in 1964 (Kuschinsky and Lüllmann 1964). The 18th and last edition was published in 2016 (Lüllmann et al. 2016). The first edition of the textbook *Allgemeine und Spezielle Pharmakologie und Toxikologie* (Karow) was published in 1993 and was not available (Karow and Lang 1993). The 33rd edition was published in 2024 (Karow and Lang-Roth 2024). All editions of the textbook *Allgemeine und Spezielle Pharmakologie und Toxikologie* were available with 3 exceptions only: The 1 st, 8th and 21 st editions could not be included in the analysis as they were not available. Further, specialist information, AVR (Drug Prescription Report), Rote Listen (red lists), guidelines, PubMed, learning platforms via medici and Amboss were analyzed. Current guidelines starting from 2018 have been selected as well.

By analyzing all available editions of the three German textbooks and the USA textbook the following categories were identified: *indications, dosages*, *ADRs, presentation* and *importance* of the drug. Codes in form of individual numerical numbers were defined for each category. The code of the respective category was used to compare the different editions of the textbooks themselves and the different German textbooks to each other. For this, a huge table has been created. The same categories and codes were used to analyze whether there are international differences. The comparison was performed between the different editions of Goodman & Gilman and between Goodman & Gilman and the German textbook (Aktories). The German textbooks were used to collect and analyze the data of dihydralazine, while the book from the USA was used to collect and analyze the data of the drug hydralazine.

The textbooks were interpreted based on studies (Table 1). The following search terms were used to select suitable studies for interpretation: “hydralazine”, “dihydralazine” and “toxicity”. The following filters were used: “Full Text”, “Clinical Study”, “Clinical Trial”, “Randomized Controlled Trial” and “Review”. The selected studies were presented in form of a traffic light scheme. An international comparison of dihydralazine in Germany and hydralazine in the USA was carried out, because dihydralazine is not available in the USA (Devarbhavi et al. 2018, 10.1016/B978-0-323-37591-7.00056-2). This comparison is possible, because dihydralazine and hydralazine are almost *identical vasodilators* (Reifert and Bussmann 1986). ADRs of the textbooks were compared with the specialist information. For the data analysis of the Rote Liste (Red List), one Rote Liste was selected per decade (from 1959 to 2025). Based on the data, again a table was created with appropriate *categories* and codes to determine changes to the drug dihydralazine over the years.

**Table 1:**
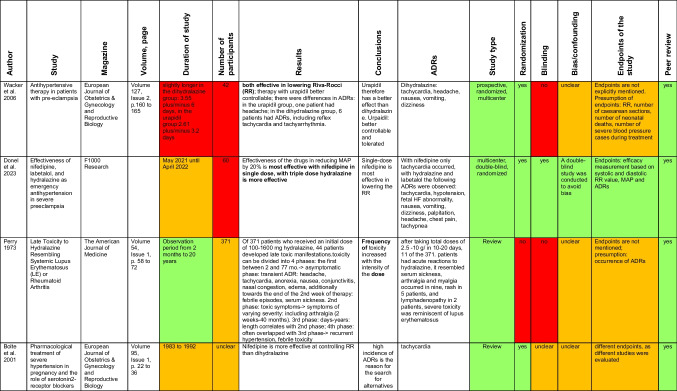
Overview of studies that were used to evaluate the textbooks

Studies were presented using a traffic light scheme with the following meaning in terms of scientific quality: green - high, yellow - not assignable, red - low

### Analysis of German and USA specialist information

The selected German specialist information was examined with regard to ADRs of dihydralazine and the result of the evaluation was compared with the three German textbooks. In contrast, the English information for healthcare professionals on hydralazine was compared with Goodman & Gilman with regard to ADRs. The results of these comparisons were first summarized in form of tables. These tables were then converted into the two diagrams (Figs. 5 and 7).

### Analysis of dihydralazine prescriptions

The number of prescriptions for the drug dihydralazine was analyzed from 1998 to 2023. The AVRs (Drug Prescription Reports) used to analyze the data (1998 to 2023) are listed in Table S3 (Schwabe and Paffrath 1999, 2000, 2001a, 2001b, 2003, 2004, 2005, 2006, 2007, 2008a, 2008b, 2009, 2010, 2011, 2012, 2013, 2014, 2015, 2016; Schwabe et al. 2017, 2018, 2019; Schwabe and Ludwig 2020; Ludwig et al. 2021, 2022, 2023). The data evaluation is intended to show whether the prescriptions of dihydralazine are declining. The prescriptions were given in defined daily dose (DDD). The costs of dihydralazine were also recorded and evaluated over time.

### Analysis of learning platforms via medici and Amboss and analysis of PubMed

Amboss (https://www.amboss.com/de, accessed 08.03.2025) and via medici (https://viamedici.thieme.de/, accessed 08.03.2025) were selected to analyze the representation of dihydralazine on learning platforms. It was investigated whether dihydralazine is still relevant on the learning platforms and whether it appears in IMPP questions. It was determined whether dihydralazine is included in the IMPP list of medicinal products to see how relevant dihydralazine is in student teaching (https://www.impp.de/pruefungen/allgemein/gegenstandskataloge.html, accessed 8.03.2025). The number of studies on the drug dihydralazine on PubMed were analyzed in chronological order (https://pubmed.ncbi.nlm.nih.gov/?term=dihydralazine, accessed 17.12.2024). The corresponding search term is “dihydralazine”. No filters were used.

### Analysis of the frequency of drug-induced lupus erythematosus

Various studies were used for the frequency analysis of lupus erythematosus (LE) caused by hydralazine (Cameron and Ramsay 1984; Iyer et al. 2017; Timlin et al. 2019; Katz and Goddard 2010). The search terms “lupus syndrome” and “hydralazine lupus syndrome” were used to analyze the drug-induced Lupus syndrome. The following filters were used: “Full Text”, “Clinical Study”, “Clinical Trial”, “Randomized Controlled Trial” and “Review”. The search leads to the following studies:

- The Lupus syndrome induced by hydralazine: a common complication with low dose treatment (https://pmc.ncbi.nlm.nih.gov/articles/PMC1442447/)
- Hydralazine Induced Lupus Syndrome Presenting with Recurrent Pericardial Effusion and a Negative Antinuclear Antibody (https://pubmed.ncbi.nlm.nih.gov/28194293/)
- Clinical Characteristics of Hydralazine-induced Lupus (https://pmc.ncbi.nlm.nih.gov/articles/PMC6707822/)
- Drug-induced Lupus: an update (https://pubmed.ncbi.nlm.nih.gov/20656071/)

### Analysis of the Rote Liste (Red List)

The Red List was analyzed to show how the indications for dihydralazine have changed over time. It also shows which ADRs have been added over time. Table S2 shows the Rote Liste (Red List) used for the analysis (Rote Liste 1959, 1967, 1975, 1985, 1994, 2005, 2018, 2024) (https://www.rote-liste.de/, accessed 20.04.2025).

### Analysis of guidelines on the use of dihydralazine/hydralazine

To analyze how dihydralazine/hydralazine is presented in guidelines, various current guidelines were selected. The following guidelines on hypertension, hypertensive pregnancy disorders and chronic heart failure (CHF) were selected:

- “Hypertonie” from the Nationalen Versorgungs-Leitlinie (2023) (https://register.awmf.org/assets/guidelines/nvl-009k_S3_Hypertonie_2023-06.pdf.)
- “Pocket Guidelines for the management of elevated blood pressure and hypertension” of European Society of Hypertension (2024) (https://www.escardio.org/Guidelines/Clinical-Practice-Guidelines/Elevated-Blood-Pressure-and-Hypertension.)
- “Hypertension” of CLINICAL PRACTICE GUIDELINES (2020) (https://www.ahajournals.org/doi/epub/10.1161/HYPERTENSIONAHA.120.15026)

- “Hypertensive Schwangerschaftserkrankungen: Diagnostik und Therapie” a Leitlinienprogram from the deutschen Gesellschaft für Gynäkologie und Geburtshilfe (DGGG), from the Österreichischen Gesellschaft für Gynäkologie und Geburtshilfe (OEGGG) and from the Schweizerischen Gesellschaft für Gynäkologie und Geburtshilfe (SGGG) (2018) (https://register.awmf.org/assets/guidelines/015-018l_S2k_Diagnostik_Therapie_hypertensiver_Schwangerschaftserkrankungen_2019-07.pdf.)
- ESC Guidelines for the diagnosis and treatment of acute and chronic heart failure McDonagh et al. 2021 (https://academic.oup.com/eurheartj/article/42/36/3599/6358045.)

## Results and discussion

### Change in the number of indications over time in Germany

**Fig. 2:**
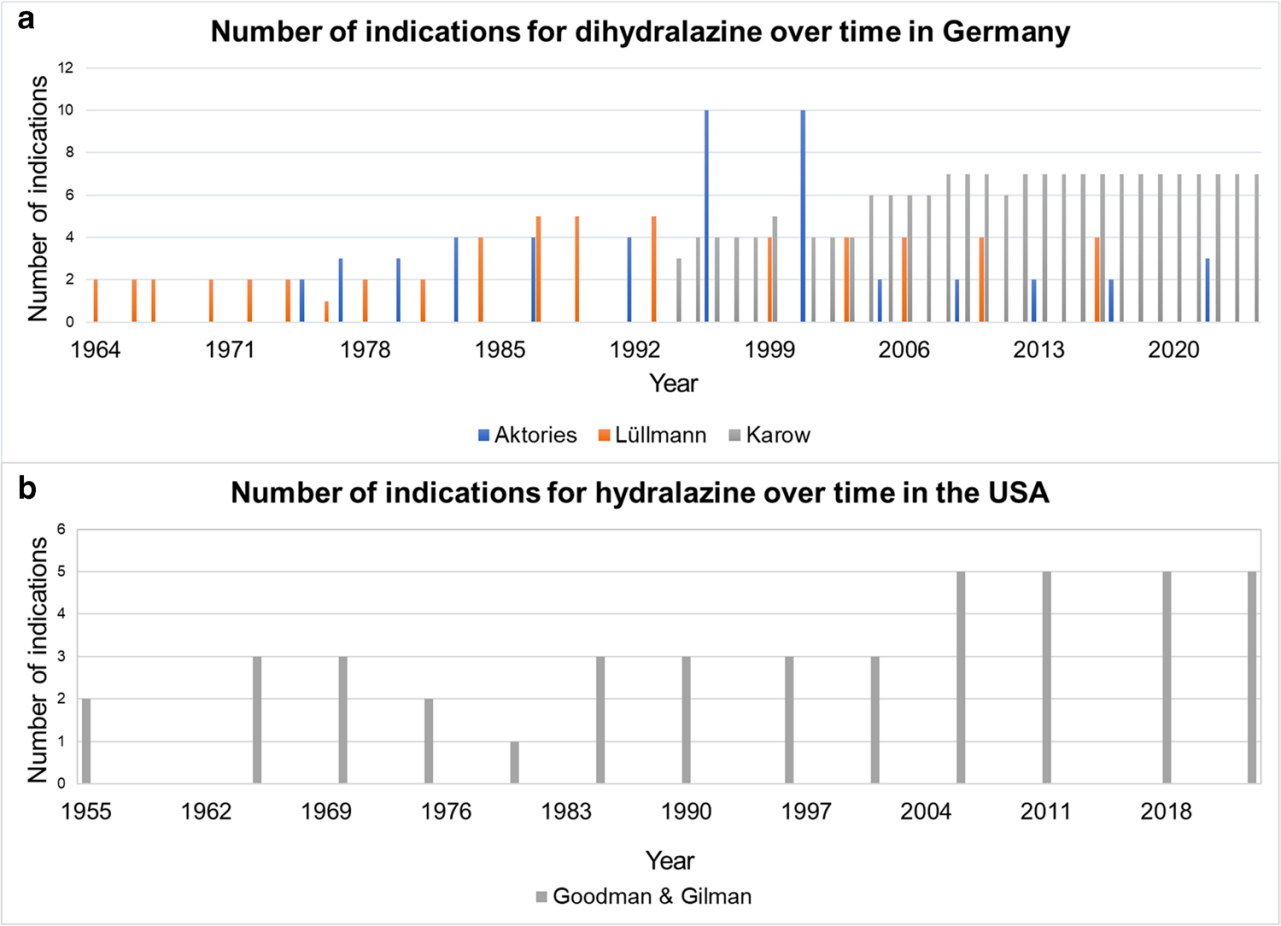
**a** and **b** Illustration of the absolute numbers of indications in three different German pharmacology textbooks and in the USA based on the textbook Goodman & Gilman

Figure 2a shows the change in the number of indications over time in Germany. There was no overall increase in the areas of application of dihydralazine over time (Fig. 2a). In the textbook *Allgemeine und spezielle Pharmakologie und Toxikologie* (Aktories), there is a particularly strong decrease in the number of areas of application from 1996 to 2022. In 1996 (7th edition), dihydralazine was described for hypertension, CHF, gestational hypertension, bradycardia, myocardial insufficiency, diabetes, hypercholesterolemia, circulatory disorder, obstruction and erectile dysfunction (Forth et al. 1996). In 2022 (13th edition), only hypertensive emergency, hypertension and CHF in African Americans were still mentioned as areas of application (Aktories et al. 2022). Table S4 illustrates the change in the number of indications and the nature of the change over time in Germany. Figure 2a also illustrates that the German textbooks are very heterogeneous. A different number of indications can be seen despite the same year of publication. In the textbook *Allgemeine und Spezielle Pharmakologie und Toxikologie* (Karow), 4 areas of application were mentioned in 1996 (4th edition) (Karow and Lang 1996). The indications mentioned include hypertension, gestational hypertension, hypertensive emergencies and gestosis (Karow and Lang 1996). Instead, 10 applications are listed in the textbook *Allgemeine und spezielle Pharmakologie und Toxikologie* (Aktories) in 1996 (7th edition).

### Change in the number of application areas over time in the USA

Figure 2b shows the change over time in the areas of application in Goodman & Gilman (USA). The analysis of the data shows an increase in the areas of application over time in contrast to Germany, where a decrease over time can be observed. Nevertheless, hydralazine has also lost importance in the USA, as has dihydralazine in Germany (Brunton et al. 2006).

### Temporal change in the areas of application in Germany

**Fig. 3:**
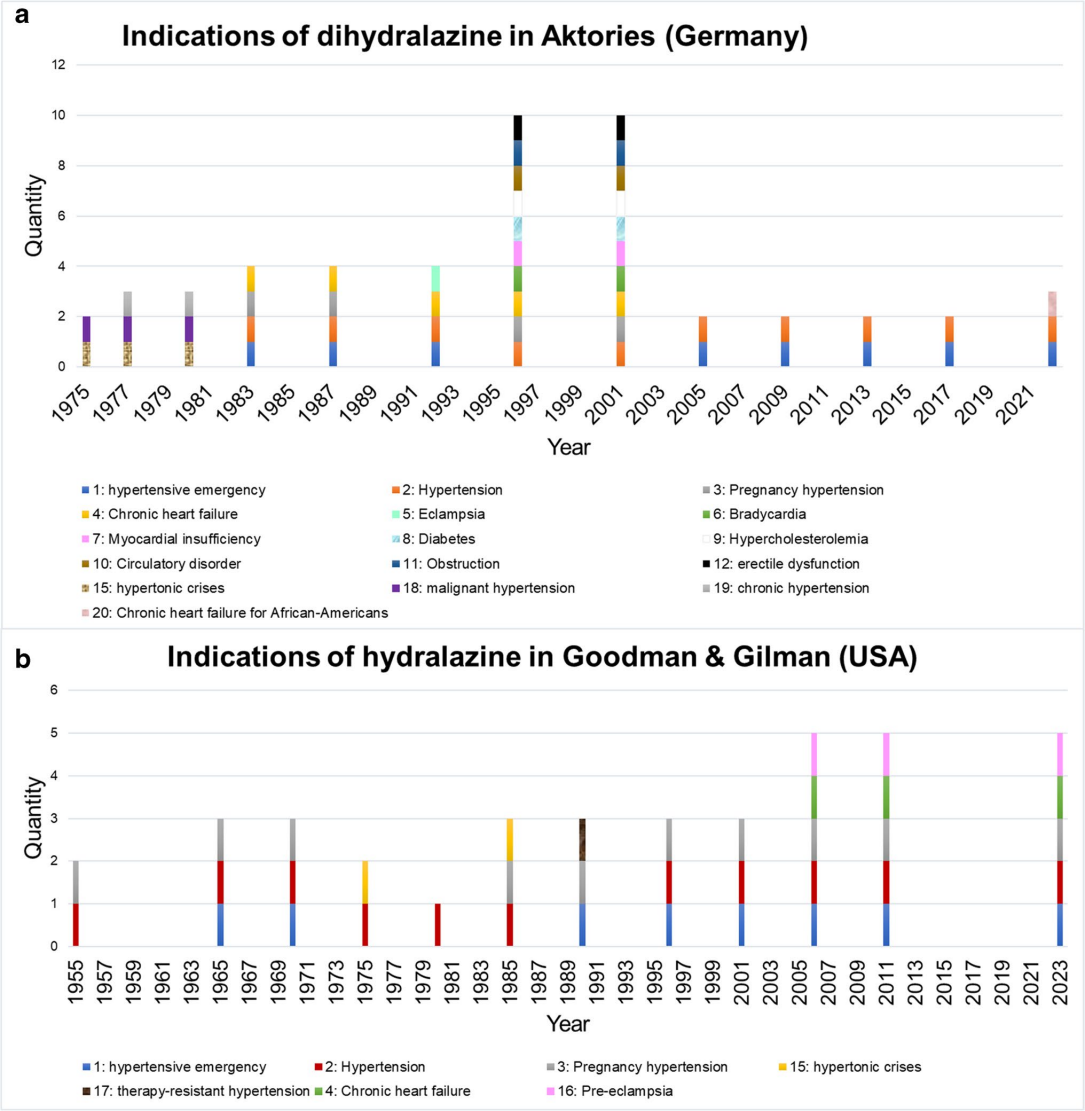
**a** and **b** Information on the indications. The absolute number is indicated

Figure 3a illustrates the areas of application of dihydralazine in the textbook (Aktories) over the period from 1975 to 2022. A distinction was made between acute and chronic indications. In 1975, malignant hypertension and hypertensive crisis were named as indications for hypertension (Forth et al. 1975). Malignant hypertension is an acute form of high blood pressure and the hypertensive crisis is also acute (Bopp et al. 2021). In 1977 (2nd edition), chronic hypertension was listed as a further indication for dihydralazine (Forth et al. 1977). The acute indication of hypertensive emergency was listed in Aktories’ textbook from 1983 (4th edition) onwards. In 1996 (7th edition) and 2001 (8th edition), pregnancy hypertension was listed as an indication. Looking at the chronological development of dihydralazine in relation to the indication of gestational hypertension (Fig. 3a), the drug played a role until the turn of the millennium, when it was no longer mentioned in the textbooks. This can be linked to existing studies that emphasize the role of alternative drugs (Wacker et al. 1998). This finding is also reflected in today’s guideline for the treatment of hypertensive disorders in pregnancy, which originates from a consensus-based statement (https://register.awmf.org/assets/guidelines/015-018l_S2k_Hypertensive-Erkrankungen-Schwangerschaft-HES-Diagnostik-Therapie_2024-07.pdf). These statements support the assumption, based on the observed circumstances, why dihydralazine is no longer mentioned in the analyzed textbook from 2001 onwards. An extension of the range of indications for dihydralazine appeared in 1983, as can be seen from the textbook (Aktories, 4th edition). The indication CHF was also listed. One possible explanation for the inclusion of this indication would be the occurrence of positive studies in connection with this disease (Morand et al. 1979). However, a decade later, Packer (1988) concluded that dihydralazine did not provide too much clinical benefit in CHF patients. This and the fact that other drugs with a better risk–benefit ratio came onto the market support the fact that dihydralazine has faded into the background for the period 2005–2017. Particularly, the class of Angiotensin-converting enzyme (ACE) inhibitors displaced the importance of vasodilators, including dihydralazine (Aktories, 10th edition) (Cardio Card Chronische Herzinsuffizienz 2023, https://leitlinien.dgk.org/files/09_2024_cardiocard_herzinsuffizienz_focused_update_2023.pdf). In 2022 (13th edition), CHF in African Americans is listed as a new indication. This indication therefore only refers to a subpopulation. In summary, dihydralazine was already used for acute and chronic forms of hypertension in 1975. The CHF indication was listed again in 2022 (13th edition), but only for African Americans.

### Temporal change in the areas of application in the USA

Figure 3b shows the change in the areas of application of hydralazine in the USA over time. The textbook Goodman & Gilman shows that hydralazine was already used for hypertension in 1955. In 1955 (2nd edition), hydralazine was used for gestational hypertension in the USA (Goodman and Gilman 1955). Compared to 1955, the latest edition of Goodman & Gilman (14th edition, 2023) lists further indications. The acute indications listed include preeclampsia and hypertensive emergencies. CHF as a chronic indication was also listed in 2023 (Brunton and Knollmann 2023).

**Fig. 4:**
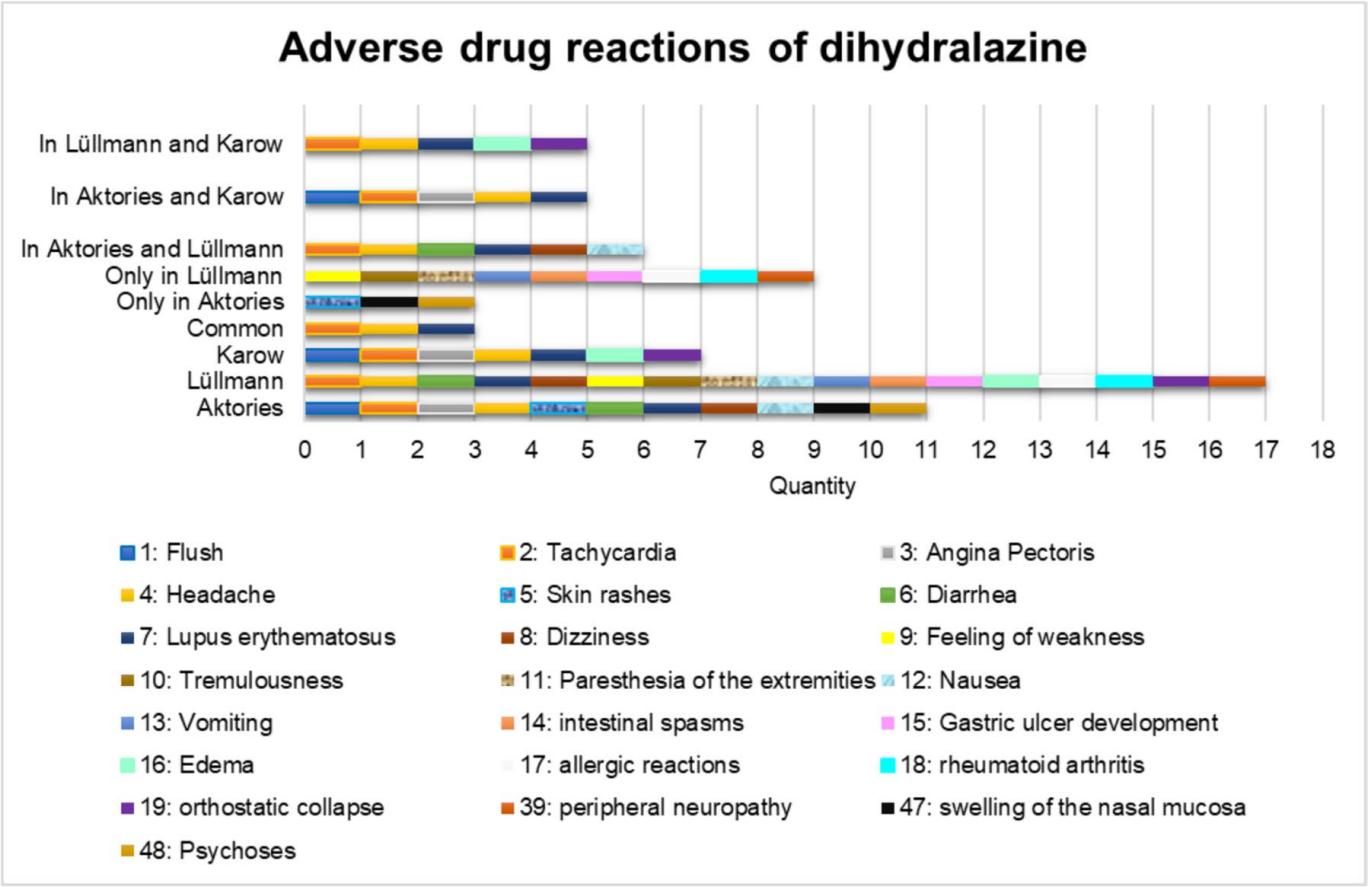
Overview of ADRs in German pharmacology textbooks

From 2006 to 2023, hydralazine was used for CHF. The late mention of hydralazine, only since 2006, could have the following reason: The important ACE inhibitor and β-adrenoreceptor antagonist studies (CONSENSUS and SOLVD) (https://www.researchgate.net/figure/CONSENSUS-and-SOLVD-Effect-of-ACE-inhibition-on-mortality_fig4_6602112) from the 1980s/1990s led to these drugs becoming a central component of CHF therapy, while vasodilators such as hydralazine were long considered reserve or outdated (https://www.abcheartfailure.org/wp-content/uploads/articles_xml/2764-3107-abchf-004-01-e20240019/2764-3107-abchf-004-01e20240019.pdf#:~:text=The%20primary%20representation%20of%20these,mechanisms%2C%20and%20recommendations%20for%20the). Older combination drugs were often “overlooked and forgotten” (Ferandes-Silva et al. 2024), despite the existence of guideline recommendations. Hydralazine only regained attention after the African-American Heart Failure Trial (A-HeFT) (Franciosa et al. 2002) and its integration into the guidelines from the mid-2000s. In this study, the combination Hydralazine/ISDN was investigated in African Americans with severe HF. This combination improved survival in addition to standard therapy (ACE inhibitors/ARBs, β-adrenoreceptor antagonist, NKCC or NCC inhibitor). In the subsequent guidelines, this combination was added as a treatment of choice alongside standard therapy in African Americans with heart failure with reduced ejection fraction (HFrEF) (V-HeFT II, Wiki Journal Club, https://www.wikijournalclub.org/wiki/V-HeFT_II#:~:text=,symptomatic%20HFrEF%20who%20cannot%20be., accessed 10.05.2025). Hydralazine was therefore only mentioned more frequently as a recommended option in more recent updates.

In Germany, dihydralazine is only used for CHF in African Americans (Aktories et al. 2022). In the previous years (2005 to 2017), dihydralazine was no longer used for CHF in the EU (Aktories et al. 2005 to 2017). This is a cross-cultural difference.

In the USA, hydralazine was still being used for gestational hypertension in 2023 (Brunton and Knollmann 2023). Pregnancy hypertension was no longer mentioned in the textbook *Allgemeine und spezielle Pharmakologie und Toxikologie* (Aktories) (Aktories et al. 2022).

### Ethnic perspective of “BiDil” (Hydralazine and Isosorbide dinitrate)

In the textbook (Aktories), the indication of CHF in African Americans was mentioned (Aktories et al. 2022). This aspect, the explicit mention of a “population”, is the reason for highlighting the ethnic perspective. “BiDil” has been or is being used to treat heart failure in African Americans (Brunton et al. 2018). “BiDil” is a trade name (BiDiL, https://bidil.com/, accessed 07.05.2025). It is a combination of ISDN and hydralazine (Brody and Hunt 2006). “BiDil” was approved in the USA in 2005 (Brody and Hunt 2006). Both drugs (Hydralazin and ISDN) have been on the market for a long time (Gen-ethisches Netzwerk e.V.). It was the first drug to be approved only for a specific ethnic group, namely people who self-identify as “black” (Gen-ethisches Netzwerk e.V.). The problem with “BiDil” is its association with racism in medicine (Gen-ethisches Netzwerk e.V.). The CDC website mentions the term"races" (CDC Heart Disease (2024), https://www.cdc.gov, accessed 14.03.2025). CDC Heart Disease is a website that deals with heart diseases in the United States. The CDC is the abbreviation for Center for Disease Control and Prevention (CDC´s division for heart disease and stroke prevention (2024), https://www.nhlbi.nih.gov/healthy-hearts-network-partner-spotlight/cdcs-division-heart-disease-and-stroke-prevention, accessed 07.05.2025). The term race is also used in the ESC guideline (McDonagh et al. 2021). In the book Goodman & Gilman (USA), the word race was also mentioned (Brunton et al. 2006). Doctors from the USA use terms such as race to assess risk factors for disease (https://scilogs.spektrum.de/die-sankore-schriften/black-history-month-2018-bidil-ein-blutdrucksenker-fuer-schwarze/, accessed 10.05.2025). In contrast, the terms “race” and “BiDil” are not mentioned in any of the selected German textbooks. Ethnic aspects are also not addressed in the specialist information. In the standard work from the USA,” BiDil” was printed in small print and mentioned in brackets in the 11th and 12th editions. In the 14th edition, the word “BiDil” itself was no longer mentioned (Brunton and Knollmann 2023). The change in the number of ethnopharmacological studies is shown in Fig. S3.

To summarize, the trade name “BiDil” and the word “races” are mentioned in the standard work from the USA. In addition, the term “race” is named on the CDC website and in the ESC guideline. In contrast, the term “race” and the trade name “BiDil” are not mentioned in the German textbooks.

### Change in the dosage of dihydralazine over time in Germany

In the textbook *Allgemeine und spezielle Pharmakologie und Toxikologie* (Aktories), a dosage of 20 to 100 mg/day was specified in the 4th to the 6th edition (Table S5). The dosage was reduced to 25 to 50 mg/day (editions 7 to 13).

### Change in the dosage of hydralazine over time in the USA

In the USA, the dose of hydralazine was also reduced (Table S6). In edition 2 (Goodman & Gilman), a usual dose of 100 to 400 mg/day was given (Goodman and Gilman 1955). From the 7th edition onwards, the dose was restricted. The maximum dose is up to 200 mg/day (Goodman et al. 1985). Studies suggest that the dose reduction was due to severe ADRs. This is because studies have shown that the frequency of severe ADRs is related to the dose (Perry 1973). This aspect is shown in Table 1.

### ADRs of dihydralazine in Germany

Figure 4 shows that dihydralazine has a *wide range* of ADRs. This is a major disadvantage of dihydralazine and has led to a search for alternatives (Bolte et al. 2001). The textbook (Lüllmann) shows the largest number of ADRs of the three German pharmacology books (Fig. 4). The three German textbooks have only a small overlap of ADRs. Only 3 ADRs in all three pharmacology textbooks (tachycardia, headache, LE). This shows that the textbooks set different priorities and only provide an overview of ADRs.

### Frequency of drug-induced lupus erythematosus

The 1984 study entitled “The Lupus syndrome induced by hydralazine” indicates an incidence of 6.7% after 3 years of treatment. The incidence of Lupus syndrome correlated with the dose. At a dose of 100 mg/day, the incidence was 5.4% and at 200 mg/day 10.4% (Cameron and Ramsay 1984). The second study mentioned above, entitled “Hydralazine Induced Lupus Syndrome Presenting with Recurrent Pericardial Effusion and a Negative Antinuclear Antibody”, does not refer to the duration or intensity of therapy with regard to the incidence of Lupus syndrome, but to the affected persons. According to this, 5 to 10% of patients are affected by Lupus (Iyer et al. 2017). The last study mentioned states an incidence of 5 to 8% after one year of treatment (Katz and Goddard 2010). Overall, different incidence figures are given. Mean values, intervals or dose-dependent values are given. It is clear from the above-mentioned studies that the intensity of the dose and the length of the treatment interval are decisive for the development of Lupus syndrome. Table S7 shows the mentioned frequencies of LE and presents an evaluation of the studies.

### Comparative ADRs analysis between textbooks and specialist information

**Fig. 5:**
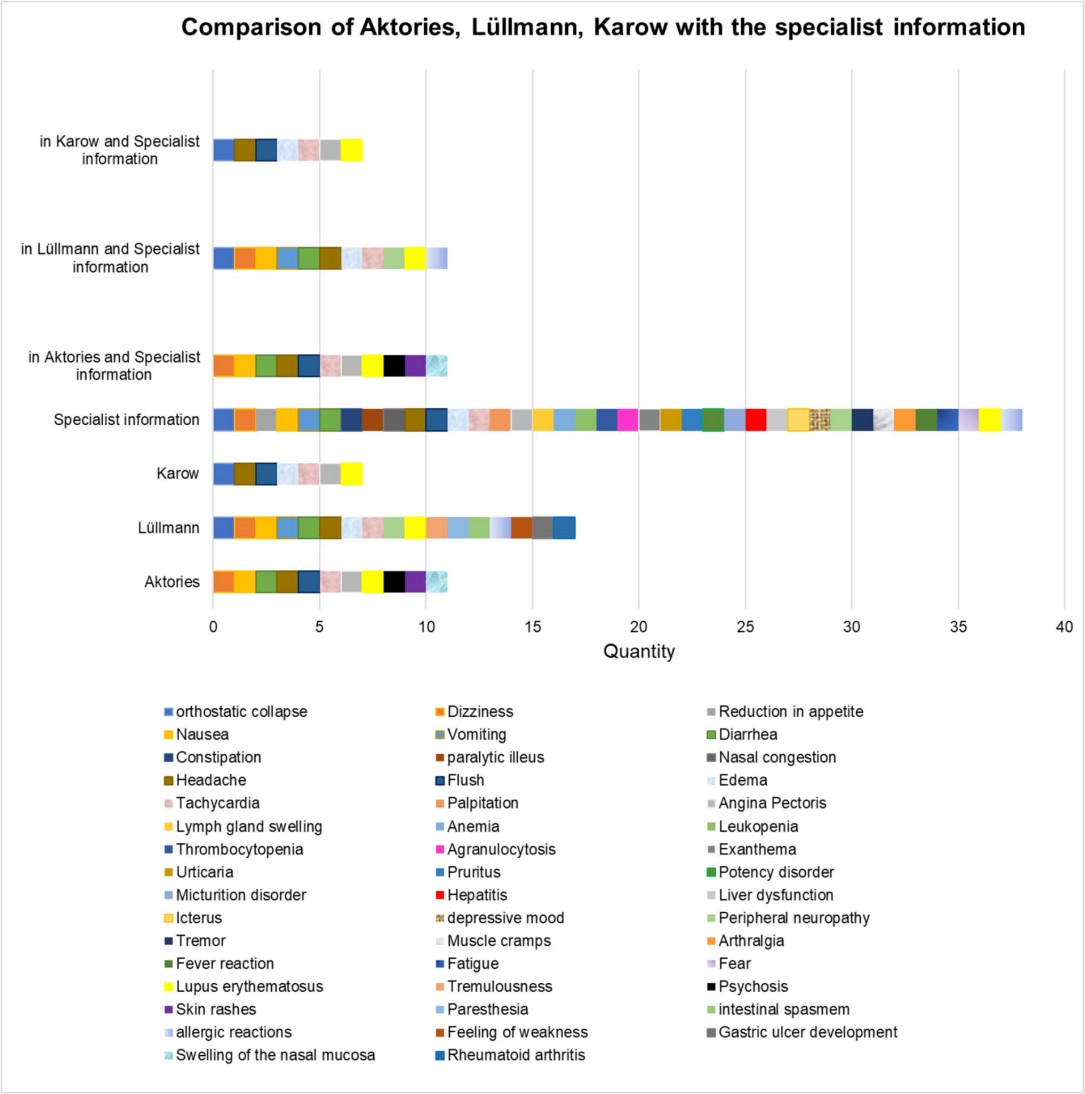
Comparison of ADRs between specialist information and textbooks

The specialist information lists significantly more ADRs (Technical information, 2001). It is more precise with regard to the data on the drug. Textbooks aim for understanding and not for completeness. Students cannot rely on a textbook alone. The lowest number of matches of ADRs is found in the specialist information with the textbook *Allgemeine und Spezielle Pharmakologie und Toxikologie* (Karow) (Fig. 5). The specialist information has 11 ADRs in common with both the textbook Pharmakologie und Toxikologie (Lüllmann) and the textbook (Aktories).

### Comparative study of ADRs between the USA and Germany

**Fig. 6:**
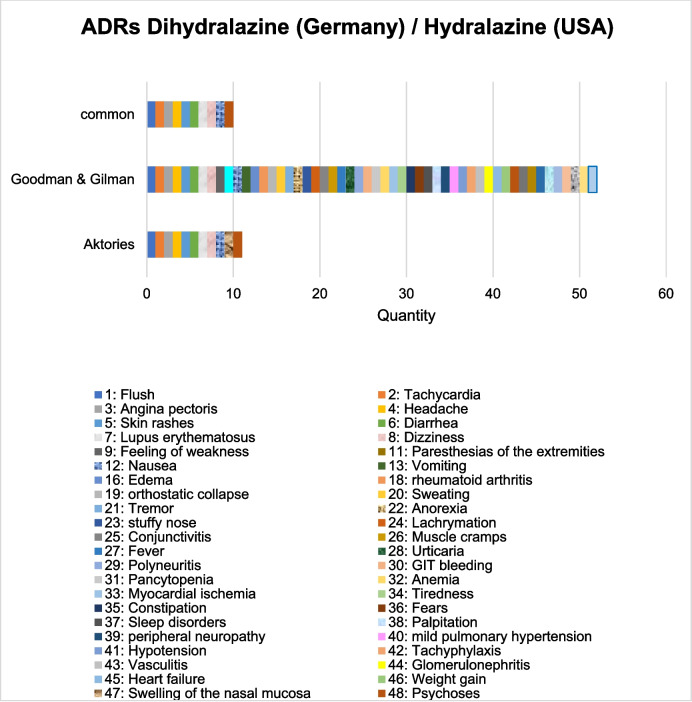
Comparison of the ADRs of dihydralazine/hydralazine

**Fig. 7:**
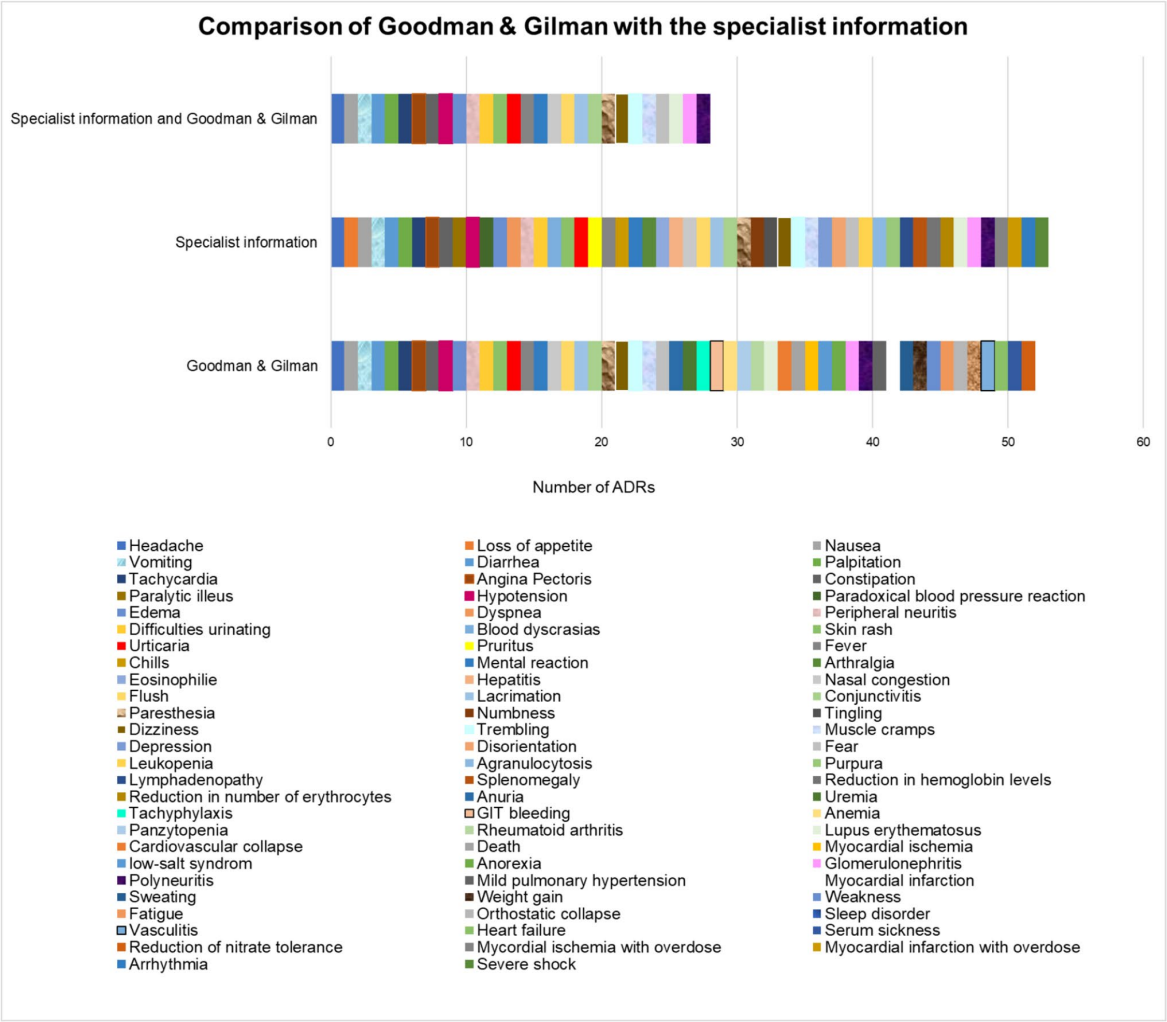
Comparison of the ADRs of hydralazine in Goodman & Gilman with a specialist information

In the Goodman & Gilman editions, all ADRs are listed in the German textbook *Allgemeine und spezielle Pharmakologie und Toxikologie* (Aktories), with one exception (swelling of the nasal mucosa). In the pharmacology book (Aktories), a total of 11 different ADRs are mentioned in the various editions (Fig. 6). The textbook from the USA lists significantly more ADRs over the years (a total of 52 different ADRs). The standard work is much more precise in this respect. This can also be seen from the similarities between the ADRs in the Goodman & Gilman book and the specialist information from the USA (Hydralazine Hydrochloride-hydralazine hydrochloride tablet, nlm.nih.gov/dailymed/lookup.cfm?setid=493a8b7e-5bb1-4db2-91a7-77ed87864131, accessed on 11.02.2025). Figure 7 shows that the specialist information from the USA has a significantly higher number of similarities between the ADRs and the textbook Goodman & Gilman than the German textbooks with the specialist information. This aspect shows how differently detailed textbooks are. In the textbook *Allgemeine und spezielle Pharmakologie und Toxikologie* (Aktories), the ADRs have not changed since the 7th edition (Forth et al. 1996). In the textbook Goodman & Gilman, the ADRs have not changed since the 13th edition (Brunton et al. 2018). The specialist information from the USA lists 53 different ADRs. This means that the standard work from the USA is up to date. Both the expert information and the pharmacology book Goodman & Gilman contain 28 ADRs in common. The English-language textbook Goodman & Gilman lists further ADRs, such as mild pulmonary hypertension, anorexia or death, which are not mentioned in the prescribing information (Fig. 7).

### Use of dihydralazine/hydralazine

As early as 1964, a combination therapy was recommended for the use of dihydralazine (Kuschinsky and Lüllmann 1964). The combination of dihydralazine and reserpine was mentioned (Kuschinsky and Lüllmann 1964). Reserpine is an alkaloid from the Indian snakeroot and acts as an antisympathomimetic drug (Misera and Seifert 2024). In 1984, the textbook *Pharmakologie und Toxikologie* refers to the antihypertensive triple combination of dihydralazine, NKCC or NCC inhibitor, and β-adrenoreceptor antagonist (Kuschinsky and Lüllmann 1984). From this it can be seen that treatment with dihydralazine was already recommended in the 1960 s with other combination partners due to the reduction of ADRs (Kuschinsky and Lüllmann 1964). Hydralazine was also used with other combination partners in the USA (Goodman and Gilman 1955). It was combined with Rauwolfia, Veratrum or Hexamethonium (Goodman and Gilman 1955). For a time, hydralazine was used together with an NKCC or NCC inhibitor and reserpine (Goodman and Gilman 1965). Nowadays, hydralazine is used like dihydralazine in Germany with an NKCC or NCC inhibitor and a β-adrenoreceptor antagonist (Fig. S4).

### Presentation of dihydralazine/hydralazine in textbooks

In the textbook *Allgemeine und spezielle Pharmakologie und Toxikologie* (Aktories), dihydralazine was consistently presented as a structural formula from the first edition (1975) to the 13th edition (2022). In the textbook *Pharmakologie und Toxikologie* (Lüllmann), dihydralazine was also presented as a structural formula in all editions. In the textbook *Allgemeine und Spezielle Pharmakologie und Toxikologie* (Karow), dihydralazine was not illustrated once. This is a clear difference to the textbooks by Aktories and Lüllmann.

In the standard work (Goodman & Gilman), hydralazine was only mentioned from the 2nd edition onwards (Goodman and Gilman 1955). From the 2nd edition (1955) to the 12th edition (2011), hydralazine was presented as a structural formula. From the 13th edition (2018) onwards, hydralazine was no longer shown pictorially (Brunton et al. 2018). This shows that hydralazine no longer plays a major role in the USA.

### Temporal analysis of the importance of dihydralazine in Germany

Figure S5 shows that the triple combination with dihydralazine was the drug of first choice from 1983 (4th edition) to 1987 (5th edition) (Forth et al. 1983 to 1987). From 1996 to 2022 (last edition of Aktories), dihydralazine was designated as the reserve antihypertensive agent. ACE inhibitors and Anti-angiotensin II type 1 (AT1) receptor antagonists are first-line agents (Forth et al. 2001). Dihydralazine is losing importance due to ADRs. The fact that dihydralazine can only be used as a combination therapy is also limiting the use of dihydralazine.

In the textbook *Pharmakologie und Toxikologie* (Lüllmann) (10th edition), dihydralazine was used in special cases, including renal insufficiency (Kuschinsky and Lüllmann 1984). Figure S6 shows that in 2016 (18th edition) dihydralazine was still recommended in the textbook (Lüllmann) for renal insufficiency (Lüllmann et al. 2016). This aspect clearly shows that dihydralazine is not yet an obsolete drug. In 2016, dihydralazine was still described as the drug of first choice in pregnancy (Lüllmann et al. 2016).

In the textbook *Allgemeine und spezielle Pharmakologie und Toxikologie* (9th edition), dihydralazine was no longer mentioned in pregnancy hypertension as early as 2005 (Aktories et al. 2005). In the textbook *Pharmakologie und Toxikologie* (Lüllmann), dihydralazine was only referred to as a reserve antihypertensive in 2016 (Lüllmann et al. 2016). In the textbook (Aktories), dihydralazine was already referred to as a reserve in 1996 (Forth et al. 1996). Here as well, it became clear that the textbooks are differently up to date.

The textbook *Allgemeine und Spezielle Pharmakologie und Toxikologie* (Karow) illustrates the development of dihydralazine from a drug of choice for Edema, Proteinuria, Hypertension (EPH) gestosis to a drug with limitations. In the textbook (Karow), dihydralazine was only designated as a reserve in 2022 (31st edition) (Karow and Lang-Roth 2022). The book by Aktories is the mostly up to date in this aspect. Figure S7 states, among other things, that nifedipine or urapidil are preferred in acute therapy (Karow and Lang-Roth 2010). Urapidil exhibits adrenergic receptor antagonism and serotonergic receptor agonism. Depending on the degree of exacerbation of the hypertension, the drug is administered orally (essential hypertension) or intravenously (i.v.) (hypertensive emergencies) (https://go.drugbank.com/drugs/DB12661, accessed 09.05.2025). Nifedipine is a L-type-calcium channel blocker. Depending on the dosage form, nifedipine is used in capsule form for vasospastic angina pectoris and for the chronic stable form. In delayed-release tablet form, nifedipine has the same indications and is also used for hypertension (https://go.drugbank.com/drugs/DB01115, accessed 09.05.2025). This assessment of nifedipine and urapidil is explained using Table 1 (Wacker et al. 2006; Donel et al. 2023; Bolte et al. 2001). According to studies, urapidil is easier to control and the therapeutic effect occurs immediately after intravenous administration. In addition, fewer serious ADRs occur (Wacker et al. 2006). In summary, it can be emphasized that, over time, drugs are being developed that are not only effective but also have fewer negative effects.

### Temporal analysis of the importance of hydralazine in the USA

Figure S8 shows that hydralazine has no longer been described as the drug of first choice since 2006 (11th edition) (Brunton et al. 2006). As with dihydralazine, the reasons for this are an unfavorable ADR spectrum and the introduction of new antihypertensives (Table 1). The area of application of hydralazine has increased, although its importance has decreased. In the textbook *Allgemeine und spezielle Pharmakologie und Toxikologie* (Aktories), the term 2nd choice drug was not mentioned. Instead, dihydralazine was referred to directly as a reserve antihypertensive (Forth et al. 1996).

### Analysis of the drug prescription reports (AVR) from 1998 to 2023

Figure S1 shows the prescription figures for dihydralazine in Germany over time. The number of prescriptions is shown in million DDD. The number of prescriptions has risen significantly since 2008, although it is still only a *reserve drug* (Forth et al. 1996). In 2008, prescriptions amounted to 4.7 million DDD (Schwabe and Paffrath 2009). In 2022, prescriptions amounted to 14.5 million DDD (Ludwig et al. 2023). This increase in prescriptions for dihydralazine can be attributed to the fact that some patients require reserve medication to normalize blood pressure (Pharmazeutische Zeitung 2023, https://www.pharmazeutische-zeitung.de, accessed 08.05.2025). The increase in prescriptions could be related to recent positive studies. One study shows that the use of low-dose dihydralazine could have a protective effect in chronic kidney disease and fibrosis (Tampe et al. 2017). Recent studies suggest that hydralazine may also have potential in arteriosclerosis and various nephrogenic diseases (Chang and Chen 2022).

From 1997 to 2010, the mean cost per DDD of dihydralazine hardly changed (Table S9). From 2011 to 2022, the mean net cost per DDD increased by approx. 12.86% (Table S9). The number of prescriptions is rising (Fig. S1), although prices are increasing. The increase in prescriptions of dihydralazine represents a contradiction to its evaluation as a *reserve drug*. An explanation for the increase in prescriptions could be the positive studies mentioned above.

### Analysis of the learning platforms via medici and Amboss

Dihydralazine is still asked in IMPP questions from the learning platforms via medici and Amboss. The learning content of via medici indicates that dihydralazine is no longer guideline-compliant in certain situations. Dihydralazine was no longer listed in the IMPP drug list (Nov. 2024). Via medici mentions dihydralazine in topics such as general information, pregnancy (indicates that dihydralazine is no longer guideline-compliant due to maternal ADR spectrum). In addition, dihydralazine was described as the second choice for arterial hypertension and hypertensive emergencies. Dihydralazine has lost relevance in student teaching.

### Number of results for dihydralazine on PubMed

The results on PubMed for dihydralazine have decreased significantly over time. Figure S2 shows 36 results in 1983 and only 1 result in 2024. It can also be deduced from these values that the importance of dihydralazine has decreased.

### Analysis of the Rote Liste (Red List)

**Fig. 8:**
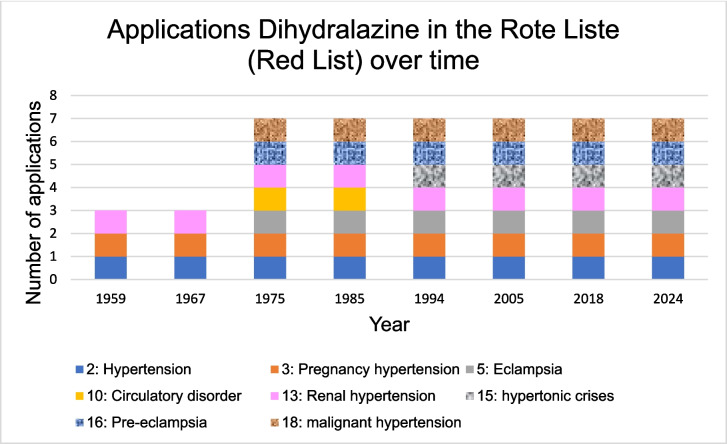
The number of applications and areas of dihydralazine in the Rote Liste (Red List) is shown. One Rote Liste (Red List) per decade was selected for this purpose

In contrast to the German textbooks, the Rote Liste (Red List) shows an increase in the use of dihydralazine over the decades (Fig. 8). Figure 8 shows that three areas of application were listed in 1959. These included hypertension, gestational hypertension and renal hypertension (Rote Liste 1959). In 1975, there was an increase to seven areas of application. New additions were malignant hypertension, eclampsia, pre-eclampsia and circulatory disorders (Rote Liste 1975). In 1994, circulatory disorders were no longer mentioned. Instead, the area of application of hypertensive crisis was listed (Rote Liste 1994). In 2024, the same areas of application were mentioned (Rote Liste 2024). The indication of hypertension has expanded over time for dihydralazine to include severe cases of hypertension. Compared to 1959, the special indications for gestational hypertension in 2024 included preeclampsia and eclampsia. Acute indications such as hypertensive crisis and malignant hypertension were also listed as indications for dihydralazine in 2024.

**Fig. 9:**
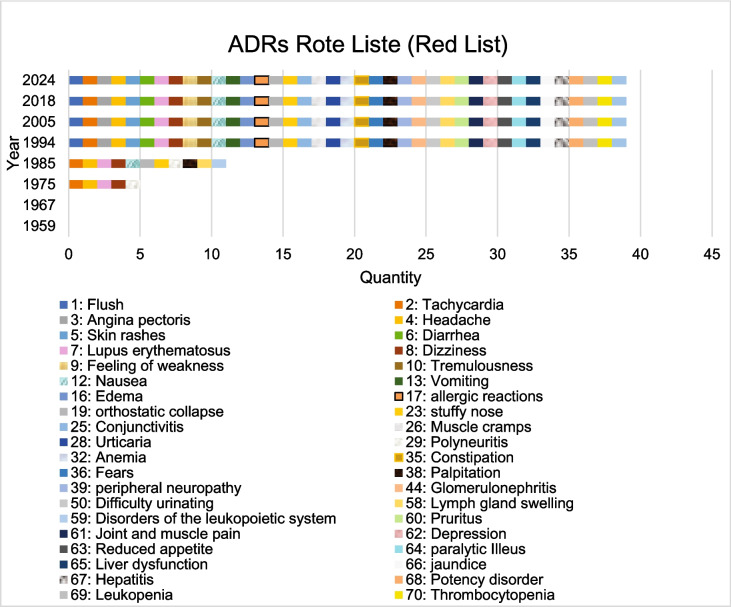
The number and type of ADRs over the years in the Rote Liste (Red List) is shown

The Rote Liste (Red List) shows an increase in ADRs over the decades (Fig. 9). No ADRs were listed in the Rote Liste of 1959 and 1967. In 1975, five ADRs were listed (headache, dizziness, tachycardia, polyneuritis and LE) (Rote Liste 1975). In 1985 there was an increase to 11 ADRs (Rote Liste 1985). In 1994, 39 ADRs were listed (Rote Liste 1994). This is more than a tripling of ADRs. In 2024, no new ADRs were added (Rote Liste 2024). With regard to the dosage of dihydralazine, a maximum dose of up to 200 mg/day was specified in 1975 (Rote Liste 1975). In 1994, a maximum dose of 100 mg/day was specified (Rote Liste 1994). In 2024, a dose of up to 100 mg/day was also specified (Rote Liste 2024). As in the pharmacology books, also here it can be seen that a dose reduction has taken place (Table S8).

In summary, the analysis shows that dihydralazine is a drug that is used today in special cases. The development of the indications demonstrates that dihydralazine is used in severe cases of hypertension. The evaluation of the Red List has shown that even a *reserve drug* such as dihydralazine can still have various indications (Fig. 8).

### Analysis of guidelines on the use of dihydralazine/hydralazine

Table 2 shows the guidelines used for the analysis**.** According to the guideline “Hypertensive Schwangerschaftserkrankungen: Diagnostik und Therapie”, a Leitlinienprogram from the deutschen Gesellschaft für Gynäkologie und Geburtshilfe (DGGG), from the Österreichischen Gesellschaft für Gynäkologie und Geburtshilfe (OEGGG) and from the Schweizerischen Gesellschaft für Gynäkologie und Geburtshilfe (SGGG) (2018), dihydralazine was not recommended for hypertensive pregnancy disorders. In the textbook (Aktories), dihydralazine has not been mentioned as an indication for pregnancy hypertension since 2005 (Aktories et al. 2005). In the textbook (Karow), dihydralazine was described as a *reserve drug* in pregnancy (Karow and Lang-Roth 2022). In the textbook (Lüllmann), dihydralazine was still described in 2016 as the drug of first choice in pregnancy (Lüllmann et al. 2016). At the same time, it was generally described as a reserve antihypertensive (Lüllmann et al. 2016). In Karow’s textbook, dihydralazine has not been described as a first-line agent in pregnancy since 2011 (Karow and Lang-Roth 2011). The textbooks by Karow and Lüllmann differ in this aspect. In the Nationale Versorgungs-Leitlinie (2023), dihydralazine was described as a reserve agent. This guideline also states that the drug classes of first choice for the treatment of hypertension include ACE inhibitors, calcium channel inhibitors and NCC- or NKCC inhibitors. The drugs of choice for arterial hypertension and the simultaneous desire to have children, on the other hand, include nifedipine as a representative of the calcium channel inhibitors, otherwise also metoprolol, as a β-adrenoreceptor antagonist, or a-methyldopa, an a_2_-adrenoceptor agonist. In Lüllmann, the use of dihydralazine in special cases was already mentioned in 1984 (Kuschinsky and Lüllmann 1984). In the textbooks by Aktories and Karow, the use in special cases was not mentioned. Hydralazine was not mentioned in the two guidelines described above. In the"Pocket guideline for the management of elevated blood pressure and hypertension"of European Society of Hypertension (2024), hydralazine should be considered for hypertension when the RR was not sufficient with a combination of three drugs and spironolactone or an additional β-adrenoreceptor antagonist and eplerenone were not sufficient. In the textbook Goodman & Gilman, hydralazine was already described as a second- and third-line hypertensive agent in 2018 (Brunton et al. 2018). In the guideline"Hypertension"of CLINICAL PRACTICE GUIDELINES (2020), only hydralazine and not dihydralazine was mentioned. In the ESC (European Society of Cardiology) 2021 guideline, hydralazine was used in combination with isosorbide dinitrate in patients who identify as black with an LVEF less than 35% or an LVEF less than 45% with a dilated left ventricle to reduce the risk of death from HF (McDonagh et al. 2021). An illustration from the ESC guideline shows that the combination therapy of isosorbide dinitrate and hydralazine was available to reduce HF-related hospitalizations/mortality for the “black race” (McDonagh et al. 2021). With regard to pregnant patients, it was mentioned that hydralazine can be used in pre-pregnancy (McDonagh et al. 2021).

**Table 2:** Overview of the status of dihydralazine/hydralazine in guidelines

Guideline	Year	Value of dihydralazine/hydralazine
**Hypertensive Schwangerschaftserkrankungen (DGGG), (OEGGG), (SGGG)**; weblink: https://register.awmf.org	2018	Mention of maternal ADRs (reflex tachycardia, headache, tachyphylaxis); according to the guideline therefore no recommendation of dihydralazine; mention of dihydralazine in DGG the treatment of severe hypertension in pregnancy; **dihydralazine has more frequent maternal ADRs than urapidil**
**CLINICAL PRACTICE GUIDLINE**; weblink: https://www.ahajournals.org	2020	Treatment of hydralazine for **severe hypertension during pregnancy**
**ESC Guidelines for the diagnosis and treatment of acute and chronic heart failure**; weblink: https://academic.oup.com	2021	Hydralazine and isosorbide dinitrate: use in patients with HF who **describe themselves as black**; use of hydralazine and isosorbide dinitrate in patients who **cannot tolerate ACE inhibitors**, among other things
**NVL**; weblink: https://register.awmf.org	2023	Designation as **reserve drug**; preferred use in dialysis patients; ADR (tachycardia)
**ESC Guidelines for the management of elevated blood pressure and hypertension**; weblink: https://www.escardio.org	2024	if RR cannot be controlled; intravenous hydralazine is **second-line therapy** for severe hypertension in pregnancy

In summary, dihydralazine has been described in Lüllmann for *special cases* since 1984. This use of dihydralazine in *special cases* was also mentioned in the NVL 2023. The current guidelines make it clear that both dihydralazine and hydralazine are used in special situations, e.g. in dialysis patients or in severe cases of hypertension. The guidelines summarize that dihydralazine/hydralazine should not be used in normal cases.

### Repurposing of hydralazine

Beyond the typical indications for hydralazine, the drug has pleiotropic effects. For example, hydralazine has antioxidant, anti-apoptotic, angiogenesis-promoting and vasoprotective properties (Chang and Chen 2022). It remains to be determined to which extent these properties are of relevance for the treatment of cardiovascular diseases.

### Limitations

The analysis is focused on the period from 1955 to 2025. Therefore, only selected pharmacology textbooks with respect to this period have been considered. The keyword index (dihydralazine, hydralazine) has been used as the basis for the evaluation of the textbooks. Thus, only the content of the pages that were assigned to the keywords has been evaluated. Only textbooks in which a chronological sequence of the evaluation of dihydralazine or hydralazine was recognizable were considered. In addition, only recent guidelines starting from 2018 onwards have been included in the analysis. Furthermore, the international comparison is limited to USA and Germany.

### Future studies

For future studies, it would be important to further investigate the mechanism of action of (di)hydralazines. (Di)hydralazines have become less important. A complete understanding of the mechanism of action would help to ensure that (di)hydralazines could be used even more specifically. In addition, it would be useful to conduct further studies on the effects of (di)hydralazines in nephrogenic diseases and arteriosclerosis, as studies indicate positive effects in these diseases.

## Conclusions

(Di)hydralazines have become less important over time. This applies to both, Germany and the USA. Nevertheless, dihydralazine is not an obsolete drug. Although dihydralazine was mentioned on the via medici and Amboss learning platforms, it was no longer included in the IMPP list of medicinal products. The number of studies on dihydralazine on PubMed has also decreased significantly. Dihydralazine is no longer the drug of choice in many areas, including pre-eclampsia, due to the large number of ADRs. However, reserve drugs such as dihydralazine must be prescribed in refractory hypertension. Hydralazine in the USA has also lost importance due to ADRs. The dosage of hydralazine in the USA and dihydralazine in Germany has been reduced. Nowadays, hydralazine in the USA is used like dihydralazine in Germany together with an NKCC or NCC inhibitor and a β-adrenoreceptor antagonist. Finally, there is potential for repurposing of hydralazine and dihydralazine.

## Supplementary Information

Below is the link to the electronic supplementary material.

ESM 1 (DOCX 720 KB)

## Data Availability

All source data for this study are available upon reasonable request.

## Abbreviations

ACE: Angiotensin-converting enzyme
ADR: Adverse drug reaction
AT1: Anti-angiotensin II type 1
AVR: Arzneiverordnungsreport (Drug Prescription Report)
CHF: Chronic heart failure
DDD: Defined daily dose
EPH: Edema (E), proteinuria (P) and hypertension (H)
GIT: Gastrointestinal tract
HF: Heart failure
HFrEF: Heart failure with reduced ejection fraction
IMPP: Institute for Medical and Pharmaceutical Examination Questions
ISDN: Isosorbide dinitrate
i.v.: Intravenously
LE: Lupus Erythematosus
mg: Milligram
NCC: NaCl cotransporter
NKCC: Na-K-2Cl cotransporter
RR: Riva-Rocci (blood pressure)
