# Supplementary material for: The change in the pharmacological significance of dihydralazine (Germany) and hydralazine (USA)
Source: Naunyn Schmiedebergs Arch Pharmacol. 2025 Jul 9;399(1):459–78. doi: 10.1007/s00210-025-04370-x (PMC12894129; doi:10.1007/s00210-025-04370-x)
Supplement: Supplementary file 1 — (DOCX 720 KB) [file 210_2025_4370_MOESM1_ESM.docx]

**The change in the pharmacological significance**

**of dihydralazine (Germany) and hydralazine (USA)**

Carina Mech and Roland Seifert

**Supplemental Tables**

**Table S1:** List of the textbooks used, with the different editions. Shown are the pages for the Key Word Index Dihydralazine/Hydralazine. Four different groups of textbooks have been examined and numbered (1 to 4) for clarity.

| **Textbook group** | **Textbook series** | **Year of publi-cation** | **Edition** | **Reference** | **Pages on Dihydralazine/Hydralazine (USA)** |
| --- | --- | --- | --- | --- | --- |
| 1 | Aktories | 1975 | 1 | Forth et al. | 126, 129, 130, 131 |
| 1 | Aktories | 1977 | 2 | Forth et al. | 119, 120, 122, 123, 124, 125, |
| 1 | Aktories | 1980 | 3 | Forth et al. | 119, 120, 122, 123, 124, 125 |
| 1 | Aktories | 1983 | 4 | Forth et al. | 143, 144, 145, 147, 148, 149 |
| 1 | Aktories | 1987 | 5 | Forth et al. | 155, 156, 159, 160, 161, 162, 281 |
| 1 | Aktories | 1992 | 6 | Forth et al. | 186, 187, 189, 190, 191, 192, 381, 382 |
| 1 | Aktories | 1996 | 7 | Forth et al. | 386, 425, 426, 428, 429, 430 |
| 1 | Aktories | 2001 | 8 | Forth et al. | 456, 501, 502, 504, 505, 506, 507 |
| 1 | Aktories | 2005 | 9 | Aktories et al. | 472, 476, 477, 479 |
| 1 | Aktories | 2009 | 10 | Aktories et al. | 421, 474, 478, 479 |
| 1 | Aktories | 2013 | 11 | Aktories et al. | 407 |
| 1 | Aktories | 2017 | 12 | Aktories et al. | 427, 428, 431, 433 |
| 1 | Aktories | 2022 | 13 | Aktories et al. | 478, 483 |
| 2 | Lüllmann | 1964 | 1 | Kuschinsky and Lüllmann | 36, 49 |
| 2 | Lüllmann | 1966 | 2 | Kuschinsky and Lüllmann | 36, 37, 49, 50 |
| 2 | Lüllmann | 1967 | 3 | Kuschinsky and Lüllmann | 36, 49 |
| 2 | Lüllmann | 1970 | 4 | Kuschinsky and Lüllmann | 37, 38, 51 |
| 2 | Lüllmann | 1972 | 5 | Kuschinsky and Lüllmann | 38, 51 |
| 2 | Lüllmann | 1974 | 6 | Kuschinsky and Lüllmann | 33, 34, 45 |
| 2 | Lüllmann | 1976 | 7 | Kuschinsky and Lüllmann | 69, 79, 80, 92 |
| 2 | Lüllmann | 1978 | 8 | Kuschinsky and Lüllmann | 75, 88, 89, 101 |
| 2 | Lüllmann | 1981 | 9 | Kuschinsky and Lüllmann | 77, 92, 93, 106, 107 |
| 2 | Lüllmann | 1984 | 10 | Kuschinsky and Lüllmann | 89, 108, 125, 126, 127 |
| 2 | Lüllmann | 1987 | 11 | Kuschinsky and Lüllmann | 111, 112, 132, 133 |
| 2 | Lüllmann | 1989 | 12 | Kuschinsky and Lüllmann | 94, 113, 114, 135, 136 |
| 2 | Lüllmann | 1993 | 13 | Kuschinsky and Lüllmann | 143, 158, 159 |
| 2 | Lüllmann | 1999 | 14 | Lüllmann and Mohr | 114, 115, 126, 127, 128 |
| 2 | Lüllmann | 2003 | 15 | Lüllmann et al. | 117, 118, 130, 131 |
| 2 | Lüllmann | 2006 | 16 | Lüllmann et al. | 154, 158, 159, 160 |
| 2 | Lüllmann | 2010 | 17 | Lüllmann et al. | 169, 174, 175, 176 |
| 2 | Lüllmann | 2016 | 18 | Lüllmann et al. | 194, 195, 200, 201 |
| 3 | Karow | 1994 | 2 | Karow and Lang | 61, 143, 151, 154 |
| 3 | Karow | 1995 | 3 | Karow and Lang | 61, 139, 140, 141, 143, 151, 154, 533b |
| 3 | Karow | 1996 | 4 | Karow and Lang | 61, 139, 140, 142, 143a, 143b, 151, 154, 533b |
| 3 | Karow | 1997 | 5 | Karow and Lang | 61, 139, 140, 142, 143 (a and b), 152, 154 |
| 3 | Karow | 1998 | 6 | Karow and Lang | 61, 139, 140, 142, 143 (a and b), 152, 154, 154 a |
| 3 | Karow | 1999 | 7 | Karow and Lang | 68, 163, 164, 166, 167, 179, 181, 182, 678 |
| 3 | Karow | 2001 | 9 | Karow and Lang | 68, 164, 165, 167, 169, 170, 184, 185, 724 |
| 3 | Karow | 2002 | 10 | Karow and Lang | 68, 163, 165, 167,169, 170, 181, 184, 185, 724 |
| 3 | Karow | 2003 | 11 | Karow and Lang-Roth | 68, 171, 173, 175, 177, 178, 192, 193, 750 |
| 3 | Karow | 2004 | 12 | Karow and Lang-Roth | 77, 78, 206, 208, 209, 225, 226, 914 |
| 3 | Karow | 2005 | 13 | Karow and Lang-Roth | 78, 79, 215, 217, 218, 972 |
| 3 | Karow | 2006 | 14 | Karow and Lang-Roth | 122, 123, 265, 266, 992, 1023 |
| 3 | Karow | 2007 | 15 | Karow and Lang-Roth | 122, 123, 263, 264, 1034, 1065 |
| 3 | Karow | 2008 | 16 | Karow and Lang-Roth | 122, 123, 263, 264, 1058, 1089, 1090 |
| 3 | Karow | 2009 | 17 | Karow and Lang-Roth | 120, 121, 259, 260, 1098, 1143, 1144 |
| 3 | Karow | 2010 | 18 | Karow and Lang-Roth | 130, 131, 270, 1136, 1183, 1184 |
| 3 | Karow | 2011 | 19 | Karow and Lang-Roth | 115, 247, 1086, 1131, 1132 |
| 3 | Karow | 2012 | 20 | Karow and Lang-Roth | 123, 251, 1096, 1143, 1144 |
| 3 | Karow | 2013 | 22 | Karow and Lang-Roth | 123, 248, 1108, 1155, 1156 |
| 3 | Karow | 2014 | 23 | Karow and Lang-Roth | 123, 246, 1106, 1153, 1154 |
| 3 | Karow | 2015 | 24 | Karow and Lang-Roth | 123, 246, 1136, 1183, 1184 |
| 3 | Karow | 2016 | 25 | Karow and Lang-Roth | 123, 246, 1152, 1199, 1200 |
| 3 | Karow | 2017 | 26 | Karow and Lang-Roth | 123, 242, 1166, 1213, 1214 |
| 3 | Karow | 2018 | 27 | Karow and Lang-Roth | 123, 242, 1188, 1239, 1240 |
| 3 | Karow | 2019 | 28 | Karow and Lang-Roth | 123, 238, 1186, 1226 |
| 3 | Karow | 2020 | 29 | Karow and Lang-Roth | 123, 238, 1198, 1238 |
| 3 | Karow | 2021 | 30 | Karow and Lang-Roth | 123, 240, 1204, 1244 |
| 3 | Karow | 2022 | 31 | Karow and Lang-Roth | 123, 234, 1198, 1240, 1241, 1242 |
| 3 | Karow | 2023 | 32 | Karow and Lang-Roth | 133, 240, 1208, 1250, 1251, 1252 |
| 3 | Karow | 2024 | 33 | Karow and Lang-Roth | 133, 240, 1212, 1258, 1259, 1260 |
| 4 | Goodman & Gilman | 1941 | 1 | Goodman and Gilman | / |
| 4 | Goodman & Gilman | 1955 | 2 | Goodman and Gilman | 756, 757, 758 |
| 4 | Goodman & Gilman | 1965 | 3 | Goodman and Gilman | 720, 721, 722, 729, 730, 731, 732 |
| 4 | Goodman & Gilman | 1970 | 4 | Goodman and Gilman | 728, 729, 730, 738, 739, 740, 741, 742 |
| 4 | Goodman & Gilman | 1975 | 5 | Goodman and Gilman | 705, 706, 707, 720, 722 |
| 4 | Goodman & Gilman | 1980 | 6 | Goodman and Gilman | 799, 800, 801, 811, 827, 1710, 1711 |
| 4 | Goodman & Gilman | 1985 | 7 | Goodman et al. | 795, 796, 800, 815, 1690 |
| 4 | Goodman & Gilman | 1990 | 8 | Gilman  et al. | 799, 800, 801, 1683 |
| 4 | Goodman & Gilman | 1996 | 9 | Hardman et al. | 15, 67, 240, 794, 795, 828, 829, 830, 1562, 1748 |
| 4 | Goodman & Gilman | 2001 | 10 | Hardman et al. | 258, 885, 886, 872, 887, 912, 1761, 1965 |
| 4 | Goodman & Gilman | 2006 | 11 | Brunton et al. | 860, 861, 862, 874, 877 ,879, 881 |
| 4 | Goodman & Gilman | 2011 | 12 | Brunton et al. | 138, 159, 779, 780, 785, 793, 795, 796, 797 |
| 4 | Goodman & Gilman | 2018 | 13 | Brunton et al. | 97, 518, 519, 522, 524, 536, 537, 544, 823 |
| 4 | Goodman & Gilman | 2023 | 14 | Brunton and Knollmann | 50, 113t, 637, 638, 641, 643, 656, 657 |

**Table S2:** Overview of the Rote Liste (Red List) used for the analysis. The pages on the index Dihydralazine were indicated.

| **Book** | **Publisher** | **Year** | **Pages to the index** |
| --- | --- | --- | --- |
| Rote Liste (Red List) | Bundesverband der Pharmazeutischen Industrie e.V. | 1959 | 556 |
| Rote Liste (Red List) | Bundesverband der Pharmazeutischen Industrie e.V. | 1967 | 802 |
| Rote Liste (Red List) | Bundesverband der Pharmazeutischen Industrie e.V. | 1975 | 18072B |
| Rote Liste (Red List) | Bundesverband der Pharmazeutischen Industrie e.V. | 1985 | 16078, D30 |
| Rote Liste (Red List) | Bundesverband der Pharmazeutischen Industrie e.V. | 1994 | 16043, 16044, D30 |
| Rote Liste (Red List) | Rote Liste Service GmBH | 2005 | 17318, 17319, D30 |
| Rote Liste (Red List) | Rote Liste Service GmBH | 2018 | 17080, 17081, D30 |
| Rote Liste (Red List) | Rote Liste Service GmBH | 2024 | 17051, 17052, D30 |

**Analysis of Arzneiverordnungsreporte (Drug prescription reports) from 1998 to 2023**

**Table S3:** List of AVRs (Drug prescription reports) used. To examine how prescribing rates and costs of dihydralazine have changed over time, the AVRs (Drug prescription reports) were analysed.

| **Book** | **Year of publication** | **Reference** |
| --- | --- | --- |
| Arzneiverordnungsreport (Drug Prescription Report) 1998 | 1999 | Schwabe and Paffrath |
| Arzneiverordnungsreport (Drug Prescription Report) 1999 | 2000 | Schwabe and Paffrath |
| Arzneiverordnungsreport (Drug Prescription Report) 2000 | 2001a | Schwabe and Paffrath |
| Arzneiverordnungsreport (Drug Prescription Report) 2001 | 2001b | Schwabe and Paffrath |
| Arzneiverordnungsreport (Drug Prescription Report) 2002 | 2003 | Schwabe and Paffrath |
| Arzneiverordnungsreport (Drug Prescription Report) 2003 | 2004 | Schwabe and Paffrath |
| Arzneiverordnungsreport (Drug Prescription Report) 2004 | 2005 | Schwabe and Paffrath |
| Arzneiverordnungsreport (Drug Prescription Report) 2005 | 2006 | Schwabe and Paffrath |
| Arzneiverordnungsreport (Drug Prescription Report) 2006 | 2007 | Schwabe and Paffrath |
| Arzneiverordnungsreport (Drug Prescription Report) 2007 | 2008a | Schwabe and Paffrath |
| Arzneiverordnungsreport (Drug Prescription Report)  2008 | 2008b | Schwabe and Paffrath |
| Arzneiverordnungsreport (Drug Prescription Report) 2009 | 2009 | Schwabe and Paffrath |
| Arzneiverordnungsreport (Drug Prescription Report) 2010 | 2010 | Schwabe and Paffrath |
| Arzneiverordnungsreport (Drug Prescription Report) 2011 | 2011 | Schwabe and Paffrath |
| Arzneiverordnungsreport (Drug Prescription Report) 2012 | 2012 | Schwabe and Paffrath |
| Arzneiverordnungsreport (Drug Prescription Report) 2013 | 2013 | Schwabe and Paffrath |
| Arzneiverordnungsreport (Drug Prescription Report) 2014 | 2014 | Schwabe and Paffrath |
| Arzneiverordnungsreport (Drug Prescription Report) 2015 | 2015 | Schwabe and Paffrath |
| Arzneiverordnungsreport (Drug Prescription Report) 2016 | 2016 | Schwabe and Paffrath |
| Arzneiverordnungsreport (Drug Prescription Report) 2017 | 2017 | Schwabe et al. |
| Arzneiverordnungsreport (Drug Prescription Report) 2018 | 2018 | Schwabe et al. |
| Arzneiverordnungsreport (Drug Prescription Report) 2019 | 2019 | Schwabe et al. |
| Arzneiverordnungsreport (Drug Prescription Report) 2020 | 2020 | Schwabe and Ludwig |
| Arzneiverordnungsreport (Drug Prescription Report) 2021 | 2021 | Ludwig et al. |
| Arzneiverordnungsreport (Drug Prescription Report) 2022 | 2022 | Ludwig et al. |
| Arzneiverordnungsreport (Drug Prescription Report) 2023 | 2023 | Ludwig et al. |

**Table S4:** shows the change in the number of indications and the type of indication in the editions of the selected pharmacology books (Aktories, Lüllmannn, Karow). Codes were selected for the indications. Each number in the table represents a different indication. The codes were defined as follows:

1 hypertensive emergency

2 Hypertension

3 Gestational hypertension

4 chronic heart failure

5 Eclampsia

6 bradycardia, bradyarrhythmia

7 Myocardial insufficiency

8 Diabetes

9 Hypercholesterolaemia

10 Circulatory disorder

11 Obstruction

12 erectile dysfunction

13 renal hypertension, poor renal perfusion

14 Gestosis

15 hypertonic crises

16 Pre-eclampsia

17 refractory hypertension

18 malignant hypertension

19 chronic hypertension

20 CHF for African Americans

| **Authors** |  | **Title** | **Year** | **Indication** |  |  |  |  |  |  |  |  |  |  | **Quantity** |
| --- | --- | --- | --- | --- | --- | --- | --- | --- | --- | --- | --- | --- | --- | --- | --- |
| Aktories, Förstermann, Hofmann, Starke |  | Allgemeine und spezielle Pharmakologie und Toxikologie | 2005 | 1 | 2 |  |  |  |  |  |  |  |  |  | 2 |
| Aktories, Förstermann, Hofmann, Starke |  | Allgemeine und spezielle Pharmakologie und Toxikologie | 2009 | 1 | 2 |  |  |  |  |  |  |  |  |  | 2 |
| Aktories, Förstermann, Hofmann, Starke |  | Allgemeine und spezielle Pharmakologie und Toxikologie | 2013 | 1 | 2 |  |  |  |  |  |  |  |  |  | 2 |
| Aktories, Förstermann, Hofmann, Starke |  | Allgemeine und spezielle Pharmakologie und Toxikologie | 2017 | 1 | 2 |  |  |  |  |  |  |  |  |  | 2 |
| Aktories, Flockerzi, Förstermann, Hofmann, |  | Allgemeine und spezielle Pharmakologie und Toxikologie | 2022 | 1 | 2 | 20 |  |  |  |  |  |  |  |  | 3 |
| Forth, Henschler, Rummel |  | Allgemeine und spezielle Pharmakologie und Toxikologie | 1975 | 18 | 15 |  |  |  |  |  |  |  |  |  | 2 |
| Forth, Rummel, Henschler |  | Allgemeine und spezielle Pharmakologie und Toxikologie | 1977 | 18 | 19 | 15 |  |  |  |  |  |  |  |  | 3 |
| Forth, Henschler, Rummel |  | Allgemeine und spezielle Pharmakologie und Toxikologie | 1980 | 19 | 18 | 15 |  |  |  |  |  |  |  |  | 3 |
| Forth, Henschler, Rummel |  | Allgemeine und spezielle Pharmakologie und Toxikologie | 1983 | 2 | 4 | 3 | 1 |  |  |  |  |  |  |  | 4 |
| Forth, Henschler, Rummel |  | Allgemeine und spezielle Pharmakologie und Toxikologie | 1987 | 1 | 2 | 3 | 4 |  |  |  |  |  |  |  | 4 |
| Forth, Henschler, Rummel, Starke |  | Allgemeine und spezielle Pharmakologie und Toxikologie | 1992 | 2 | 5 | 4 | 1 |  |  |  |  |  |  |  | 4 |
| Forth, Henschler, Rummel, Starke |  | Allgemeine und spezielle Pharmakologie und Toxikologie | 1996 | 2 | 4 | 3 | 6 | 7 | 8 | 9 | 10 | 11 | 12 |  | 10 |
| Forth, Henschler, Rummel, Starke |  | Allgemeine und spezielle Pharmakologie und Toxikologie | 2001 | 2 | 4 | 3 | 6 | 7 | 8 | 9 | 10 | 11 | 12 |  | 10 |
| Lüllmann, Mohr |  | Pharmakologie und Toxikologie | 1999 | 13 | 3 | 14 | 15 |  |  |  |  |  |  |  | 4 |
| Lüllmann, Mohr, Wehling |  | Pharmakologie und Toxikologie | 2003 | 13 | 3 | 14 | 15 |  |  |  |  |  |  |  | 4 |
| Lüllmann, Mohr, Hein |  | Pharmakologie und Toxikologie | 2006 | 13 | 3 | 14 | 15 |  |  |  |  |  |  |  | 4 |
| Lüllmann, Mohr, Hein |  | Pharmakologie und Toxikologie | 2010 | 13 | 3 | 14 | 15 |  |  |  |  |  |  |  | 4 |
| Lüllmann, Mohr, Wehling, Hein |  | Pharmakologie und Toxikologie | 2016 | 13 | 3 | 14 | 15 |  |  |  |  |  |  |  | 4 |
| Kuschinsky, Lüllmann |  | Kurzes Lehrbuch der Pharmakologie | 1964 | 2 | 10 |  |  |  |  |  |  |  |  |  | 2 |
| Kuschinsky, Lüllmann |  | Kurzes Lehrbuch der Pharmakologie | 1966 | 2 | 10 |  |  |  |  |  |  |  |  |  | 2 |
| Kuschinsky, Lüllmann |  | Kurzes Lehrbuch der Pharmakologie | 1967 | 2 | 10 |  |  |  |  |  |  |  |  |  | 2 |
| Kuschinsky, Lüllmann |  | Kurzes Lehrbuch der Pharmakologie | 1970 | 2 | 10 |  |  |  |  |  |  |  |  |  | 2 |
| Kuschinsky, Lüllmann |  | Kurzes Lehrbuch der Pharmakologie | 1972 | 2 | 10 |  |  |  |  |  |  |  |  |  | 2 |
| Kuschinsky, Lüllmann |  | Kurzes Lehrbuch der Pharmakologie | 1974 | 2 | 10 |  |  |  |  |  |  |  |  |  | 2 |
| Kuschinsky, Lüllmann |  | Kurzes Lehrbuch der Pharmakologie | 1976 | 2 |  |  |  |  |  |  |  |  |  |  | 1 |
| Kuschinsky, Lüllmann |  | Kurzes Lehrbuch der Pharmakologie | 1978 | 2 | 13 |  |  |  |  |  |  |  |  |  | 2 |
| Kuschinsky, Lüllmann |  | Kurzes Lehrbuch der Pharmakologie | 1981 | 2 | 13 |  |  |  |  |  |  |  |  |  | 2 |
| Kuschinsky, Lüllmann |  | Pharmakologie und Toxikologie | 1984 | 2 | 3 | 13 | 14 |  |  |  |  |  |  |  | 4 |
| Kuschinsky, Lüllmann |  | Pharmakologie und Toxikologie | 1987 | 15 | 3 | 13 | 2 | 14 |  |  |  |  |  |  | 5 |
| Kuschinsky, Lüllmann |  | Pharmakologie und Toxikologie | 1989 | 15 | 13 | 3 | 14 | 2 |  |  |  |  |  |  | 5 |
| Kuschinsky, Lüllmann |  | Pharmakologie und Toxikologie | 1993 | 13 | 3 | 14 | 2 | 15 |  |  |  |  |  |  | 5 |
| Karow, Lang |  | Allgemeine und Spezielle Pharmakologie und Toxikologie | 1994 | 2 | 15 | 4 |  |  |  |  |  |  |  |  | 3 |
| Karow, Lang |  | Allgemeine und Spezielle Pharmakologie und Toxikologie | 1995 | 2 | 3 | 15 | 14 |  |  |  |  |  |  |  | 4 |
| Karow, Lang |  | Allgemeine und Spezielle Pharmakologie und Toxikologie | 1996 | 2 | 3 | 1 | 14 |  |  |  |  |  |  |  | 4 |
| Karow, Lang |  | Allgemeine und Spezielle Pharmakologie und Toxikologie | 1997 | 2 | 1 | 3 | 14 |  |  |  |  |  |  |  | 4 |
| Karow, Lang |  | Allgemeine und Spezielle Pharmakologie und Toxikologie | 1998 | 2 | 3 | 1 | 14 |  |  |  |  |  |  |  | 4 |
| Karow, Lang |  | Allgemeine und Spezielle Pharmakologie und Toxikologie | 1999 | 2 | 3 | 1 | 15 | 14 |  |  |  |  |  |  | 5 |
| Karow, Lang-Ruth |  | Allgemeine und Spezielle Pharmakologie und Toxikologie | 2001 | 1 | 2 | 14 | 3 |  |  |  |  |  |  |  | 4 |
| Karow, Lang |  | Allgemeine und Spezielle Pharmakologie und Toxikologie | 2002 | 2 | 1 | 3 | 14 |  |  |  |  |  |  |  | 4 |
| Karow, Lang-Roth |  | Allgemeine und Spezielle Pharmakologie und Toxikologie | 2003 | 2 | 1 | 14 | 3 |  |  |  |  |  |  |  | 4 |
| Karow, Lang-Roth |  | Allgemeine und Spezielle Pharmakologie und Toxikologie | 2004 | 2 | 14 | 16 | 5 | 1 |  |  |  |  |  |  | 5 |
| Karow, Lang-Ruth |  | Allgemeine und Spezielle Pharmakologie und Toxikologie | 2005 | 2 | 1 | 14 | 16 | 5 | 3 |  |  |  |  |  | 6 |
| Karow, Lang-Roth |  | Allgemeine und Spezielle Pharmakologie und Toxikologie | 2006 | 2 | 1 | 14 | 16 | 5 | 3 |  |  |  |  |  | 6 |
| Karow, Lang-Roth |  | Allgemeine und Spezielle Pharmakologie und Toxikologie | 2007 | 2 | 1 | 14 | 16 | 5 | 3 |  |  |  |  |  | 6 |
| Karow, Lang-Roth |  | Allgemeine und Spezielle Pharmakologie und Toxikologie | 2008 | 2 | 15 | 1 | 14 | 5 | 16 | 3 |  |  |  |  | 7 |
| Karow, Lang-Ruth |  | Allgemeine und Spezielle Pharmakologie und Toxikologie | 2009 | 2 | 1 | 14 | 16 | 5 | 3 | 15 |  |  |  |  | 7 |
| Karow, Lang-Roth |  | Allgemeine und Spezielle Pharmakologie und Toxikologie | 2010 | 2 | 1 | 14 | 16 | 5 | 3 | 15 |  |  |  |  | 7 |
| Karow, Lang-Ruth |  | Allgemeine und Spezielle Pharmakologie und Toxikologie | 2011 | 2 | 15 | 14 | 16 | 5 | 3 |  |  |  |  |  | 6 |
| Karow, Lang-Roth |  | Allgemeine und Spezielle Pharmakologie und Toxikologie | 2012 | 2 | 15 | 1 | 14 | 5 | 16 | 3 |  |  |  |  | 7 |
| Karow, Lang-Ruth |  | Allgemeine und Spezielle Pharmakologie und Toxikologie | 2013 | 2 | 14 | 5 | 16 | 1 | 15 | 3 |  |  |  |  | 7 |
| Karow, Lang-Roth |  | Allgemeine und Spezielle Pharmakologie und Toxikologie | 2014 | 2 | 15 | 1 | 14 | 16 | 5 | 3 |  |  |  |  | 7 |
| Karow, Lang-Roth |  | Allgemeine und Spezielle Pharmakologie und Toxikologie | 2015 | 2 | 15 | 14 | 16 | 5 | 1 | 3 |  |  |  |  | 7 |
| Karow, Lang-Roth |  | Allgemeine und Spezielle Pharmakologie und Toxikologie | 2016 | 2 | 15 | 14 | 16 | 5 | 1 | 3 |  |  |  |  | 7 |
| Karow, Lang-Ruth |  | Allgemeine und Spezielle Pharmakologie und Toxikologie | 2017 | 2 | 15 | 14 | 16 | 5 | 3 | 1 |  |  |  |  | 7 |
| Karow, Lang-Roth |  | Allgemeine und Spezielle Pharmakologie und Toxikologie | 2018 | 2 | 15 | 1 | 14 | 5 | 16 | 3 |  |  |  |  | 7 |
| Karow, Lang-Ruth |  | Allgemeine und Spezielle Pharmakologie und Toxikologie | 2019 | 2 | 15 | 14 | 16 | 5 | 3 | 1 |  |  |  |  | 7 |
| Karow, Lang-Ruth |  | Allgemeine und Spezielle Pharmakologie und Toxikologie | 2020 | 2 | 15 | 14 | 16 | 5 | 3 | 1 |  |  |  |  | 7 |
| Karow, Lang-Ruth |  | Allgemeine und Spezielle Pharmakologie und Toxikologie | 2021 | 2 | 15 | 14 | 16 | 5 | 3 | 1 |  |  |  |  | 7 |
| Karow, Lang-Roth |  | Allgemeine und Spezielle Pharmakologie und Toxikologie | 2022 | 2 | 15 | 14 | 16 | 5 | 1 | 3 |  |  |  |  | 7 |
| Karow, Lang-Ruth |  | Allgemeine und Spezielle Pharmakologie und Toxikologie | 2023 | 2 | 15 | 14 | 16 | 5 | 3 | 1 |  |  |  |  | 7 |
| Karow, Lang-Roth |  | Allgemeine und Spezielle Pharmakologie und Toxikologie | 2024 | 2 | 15 | 14 | 16 | 5 | 3 | 1 |  |  |  |  | 7 |

**Table S5:** Summary of dihydralazine dosage in editions 1 to 13. Dosage data are taken from the textbook General and Special Pharmacology and Toxicology (Aktories).

| **Textbook** | **Edition** | **Dosage** |
| --- | --- | --- |
| Aktories | 1, 2 and 3 | / |
| Aktories | 4 to 6 | 20-100 mg/day |
| Aktories | 7 to 13 | 25-50 mg/day |
|  |  |  |

**Table S6:** Overview of the dosage of hydralazine in editions 2 to 14. Dosage data are taken from the textbook The PHARMACOLOGICAL BASIS OF THERAPEUTICS (Goodman & Gilman).

| **Textbook** | **Edition** | **Dosage** |
| --- | --- | --- |
| Goodman & Gilman | 2 | usual dose 100 to 400 mg/day |
| Goodman & Gilman | 3 | up to 400 mg/day, 10 to 20 mg several times a day |
| Goodman & Gilman | 4 | Usual oral dose 100-200 mg/day; 10-20 mg several times a day; up to 200 mg/day. |
| Goodman & Gilman | 5 and 6 | Max. Dose 400mg/day; 10-20mg/several times daily; usual oral dose 100-200mg/day. |
| Goodman & Gilman | 7 | up to 200mg/day; usual dose 25-100 mg 2x daily. |
| Goodman & Gilman | 8 and 9 | up to 200 mg/day; usual dose 25-100 mg twice daily. |
| Goodman & Gilman | 10 | up to 200 mg/day; usual dose 25-100 mg twice daily. |
| Goodman & Gilman | 11 | up to 200 mg/day; usual dose 25-100 mg twice daily. |
| Goodman & Gilman | 12 | up to 200 mg/day; usual dose 25-100 mg twice daily. |
| Goodman & Gilman | 14 | up to 200 mg/day; usual dose 25-100 mg twice daily. |
| Goodman & Gilman | 13 | up to 200mg/day; usual dose 25-100 mg 2x daily |

**Table S7:** Overview of studies used to analyze the frequency of lupus induced by hydralzine/dihydralazine. The traffic light scheme was also used here.

| **Author** | **Magazine** | **Frequency of lupus erythematosus** | **Duration of study** | **Number of participants** | **Study type** | **Randomization** | **Blinding** | **Bias/confounding** | **Endpoints of the study** | **Peer review** |
| --- | --- | --- | --- | --- | --- | --- | --- | --- | --- | --- |
| Cameron and Ramsay 1984 | BRITISH MEDICAL JOURNAL | after **three years of** treatment: **Incidence** of **6,7 %;** Frequency was **dose-dependent**: Incidence 5.4% at dose of 100 mg/day, at 200 mg/day 10.4% | 57 months | 281 Pat. | Follow-up study | no | no | 12 % of patients could no longer be followed: were assessed as if they had not developed lupus syndrome | are not mentioned; presumption: occurrence of lupus syndrome | yes |
| Iyer et al. 2017 | Hindawi Publishing Corporation | Frequency 5-10 % of patients (information was provided independently of the case report | not named | 1 case | Case Report | no | no | unclear | not named | yes |
| Timlin et al. 2019 | Cureus | Frequency 7-13% | over 12 months | 7 Pat. | monocentric and retrospective | unclear | unclear | unclear | Laboratory values: including positive ANA | yes |
| Katz and Goddard 2010 | ScienceDirect | relative incidence is 5-8%/year after treatment with hydralazine | not named | not named | Review | not named | not named | unclear | Combination of various studies | yes |

**Table S8:** Change in maximum daily doses over the years in the Rote Liste (Red List).

| **Book** | **Year** | **maximum dose** |
| --- | --- | --- |
| Rote Liste (Red List) | 1959 | / |
| Rote Liste (Red List) | 1967 | / |
| Rote Liste (Red List) | 1975 | 200 mg/day |
| Rote Liste (Red List) | 1985 | 200 mg/day |
| Rote Liste (Red List) | 1994 | Max. Daily doses 100 mg |
| Rote Liste (Red List) | 2005 | Max. Daily doses 100 mg |
| Rote Liste (Red List) | 2018 | Max. Daily doses 100 mg |
| Rote Liste (Red List) | 2024 | Max. Daily doses 100 mg |

**Table S9:** shows the average costs and average net costs per DDD over time. From 1997 to 2010, the AVR (Drug Prescription Report) referred to mean costs per DDD. From 2011 onwards, the average net cost per DDD was used.

| **Year** | **Average cost per DDD** | **Average net doses per DDD** |
| --- | --- | --- |
| 1997 | 0,83 € |  |
| 1998 | 0,82 € |  |
| 1999 | 0,82 € |  |
| 2000 | 0,81 € |  |
| 2001 | 0,79 € |  |
| 2002 | 0,79 € |  |
| 2003 | 0,79 € |  |
| 2004 | 0,80 € |  |
| 2005 | 0,79 € |  |
| 2006 | 0,78 € |  |
| 2007 | 0,82 € |  |
| 2008 | 0,83 € |  |
| 2009 | 0,84 € |  |
| 2010 | 0,83 € |  |
| 2011 |  | 0,70 € |
| 2012 |  | 0,70 € |
| 2013 |  | 0,72 € |
| 2014 |  | 0,76 € |
| 2015 |  | 0,75 € |
| 2016 |  | 0,75 € |
| 2017 |  | 0,75 € |
| 2018 |  | 0,75 € |
| 2019 |  | 0,75 € |
| 2020 |  | 0,76 € |
| 2021 |  | 0,77 € |
| 2022 |  | 0,79 € |

**
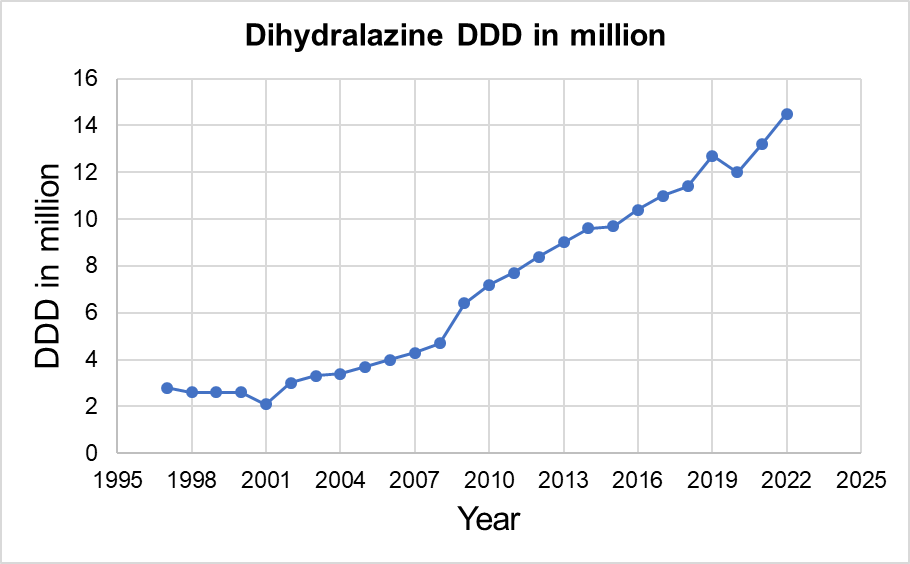
**

**Figure S1:** Information on prescriptions. The prescribed daily doses (DDD) are shown in millions. The data originate from the AVR (Drug Prescription Report) from 1998 to 2023.

**
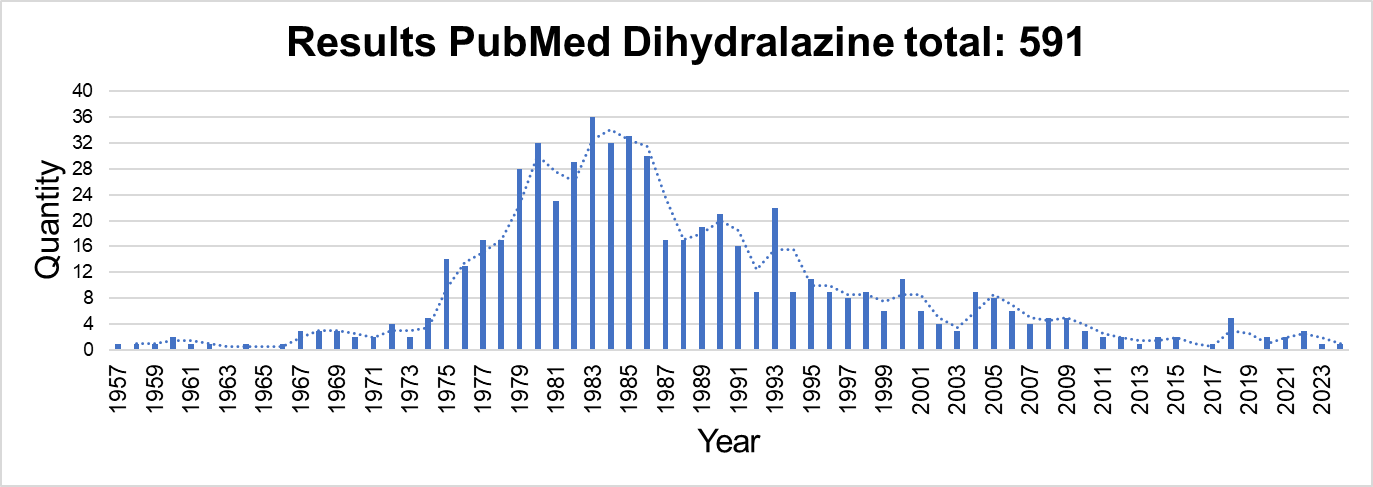
Figure S2:** Information about the number of results on PubMed for the keyword dihydralazine.

**Figure S3:** Number of ethnopharmacological studies on PubMed over time.


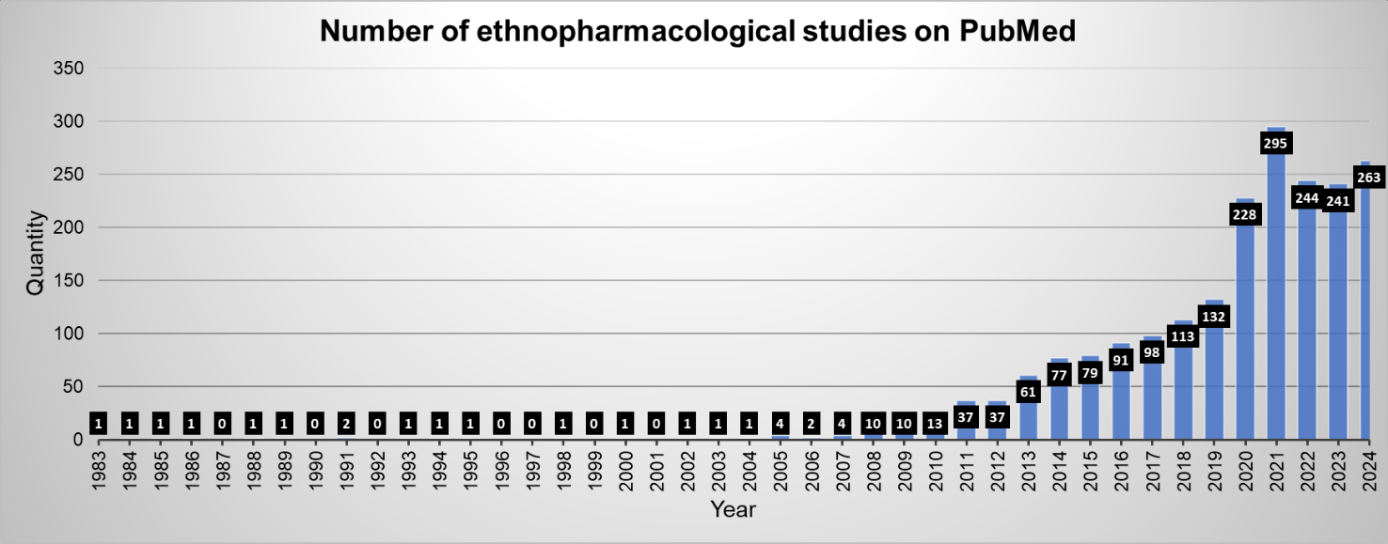


**
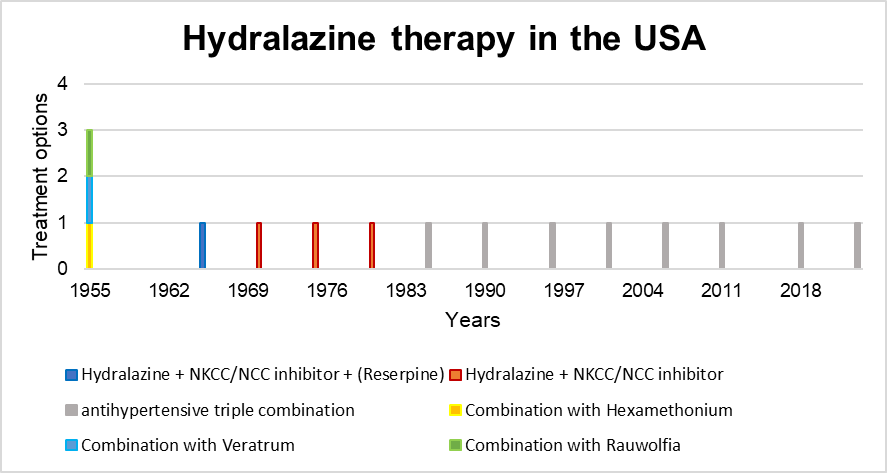
Figure S4:** Treatment options of hydralazine over time in the US based on Goodman & Gilman.


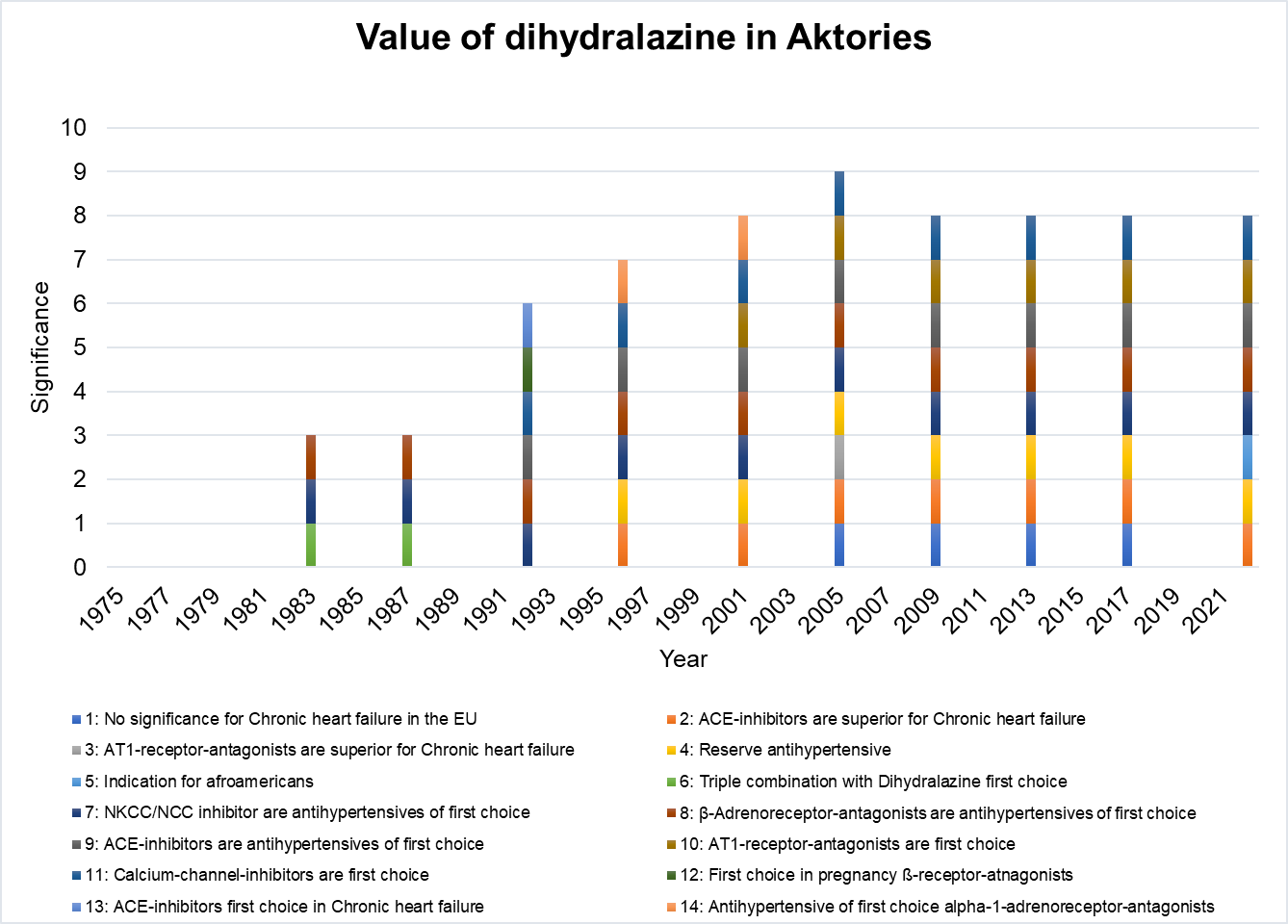
**Figure S5:** Information on the change in place value in the textbook Allgemeine und spezielle Pharmakologie und Toxikologie (Aktories).


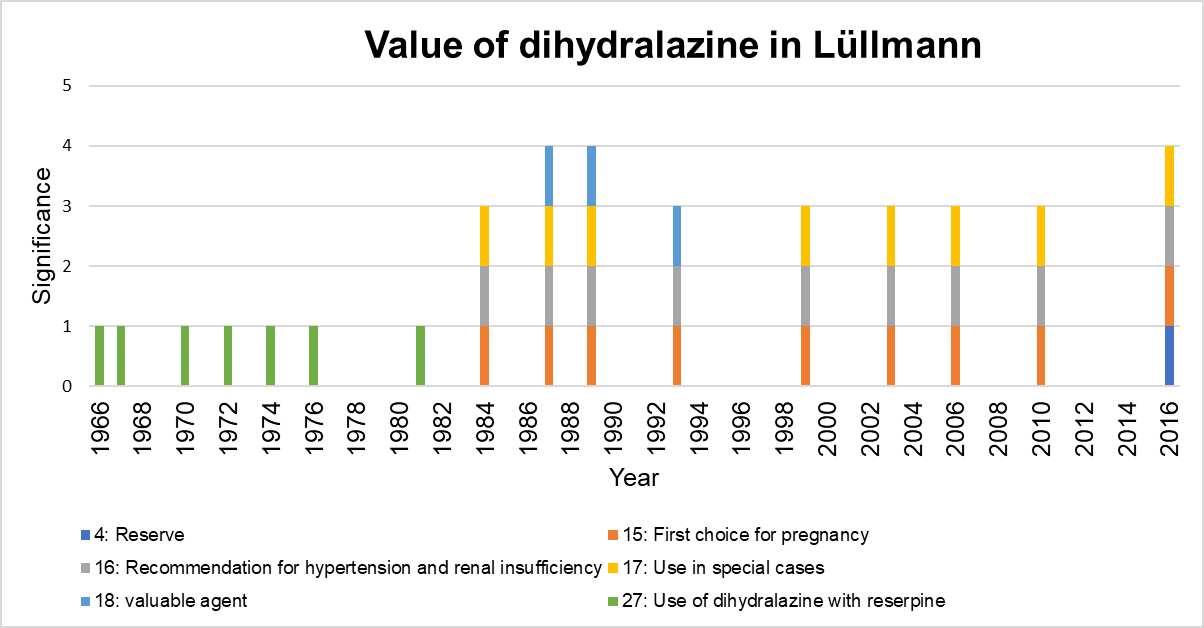
**Figure S6:** Information on the change in place value in the textbook Pharmakologie und Toxikologie (Lüllmann).


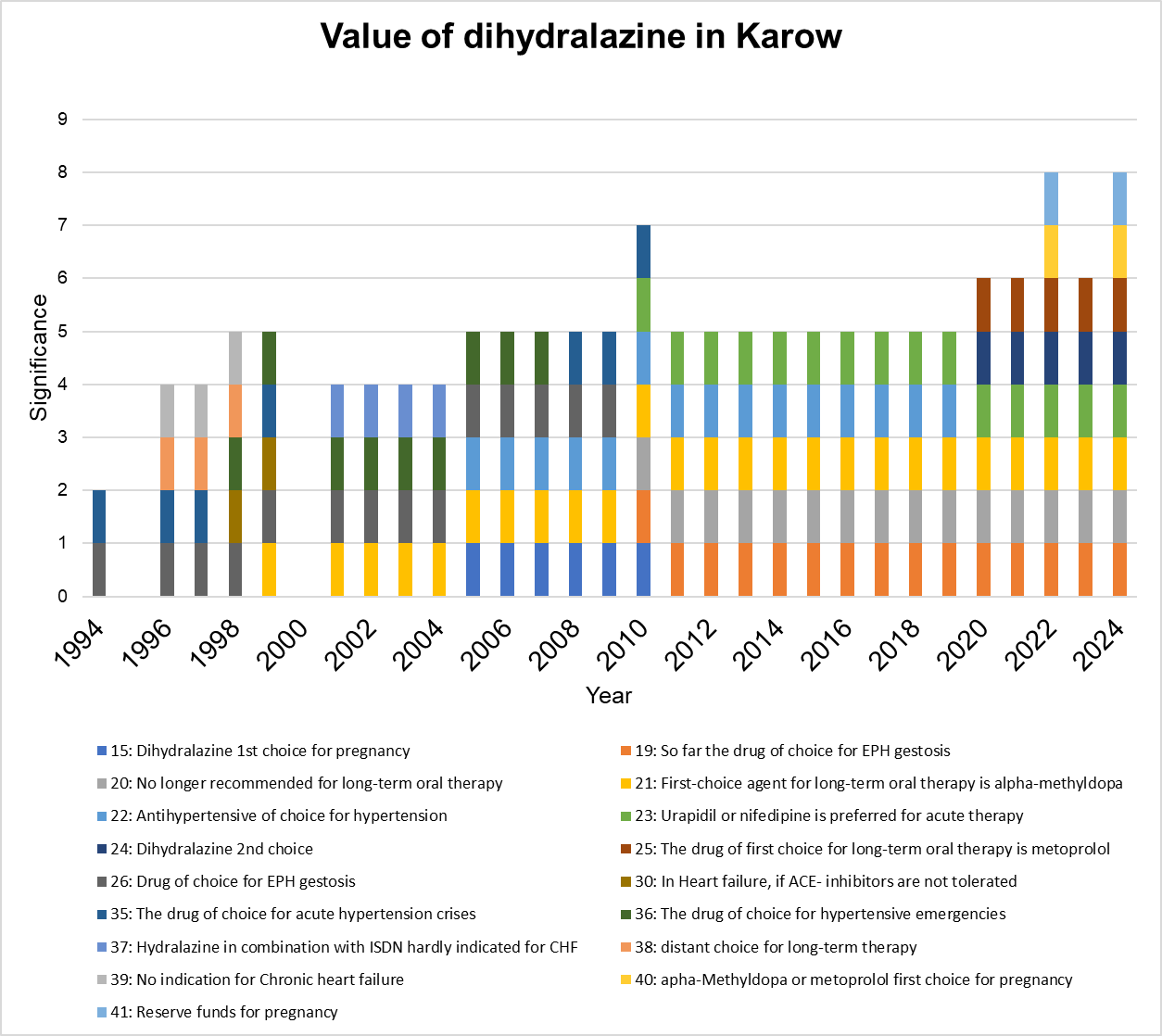
**Figure S7**: Information on the change in place value in the textbook Allgemeine und Spezielle Pharmakologie und Toxikologie (Karow).

**
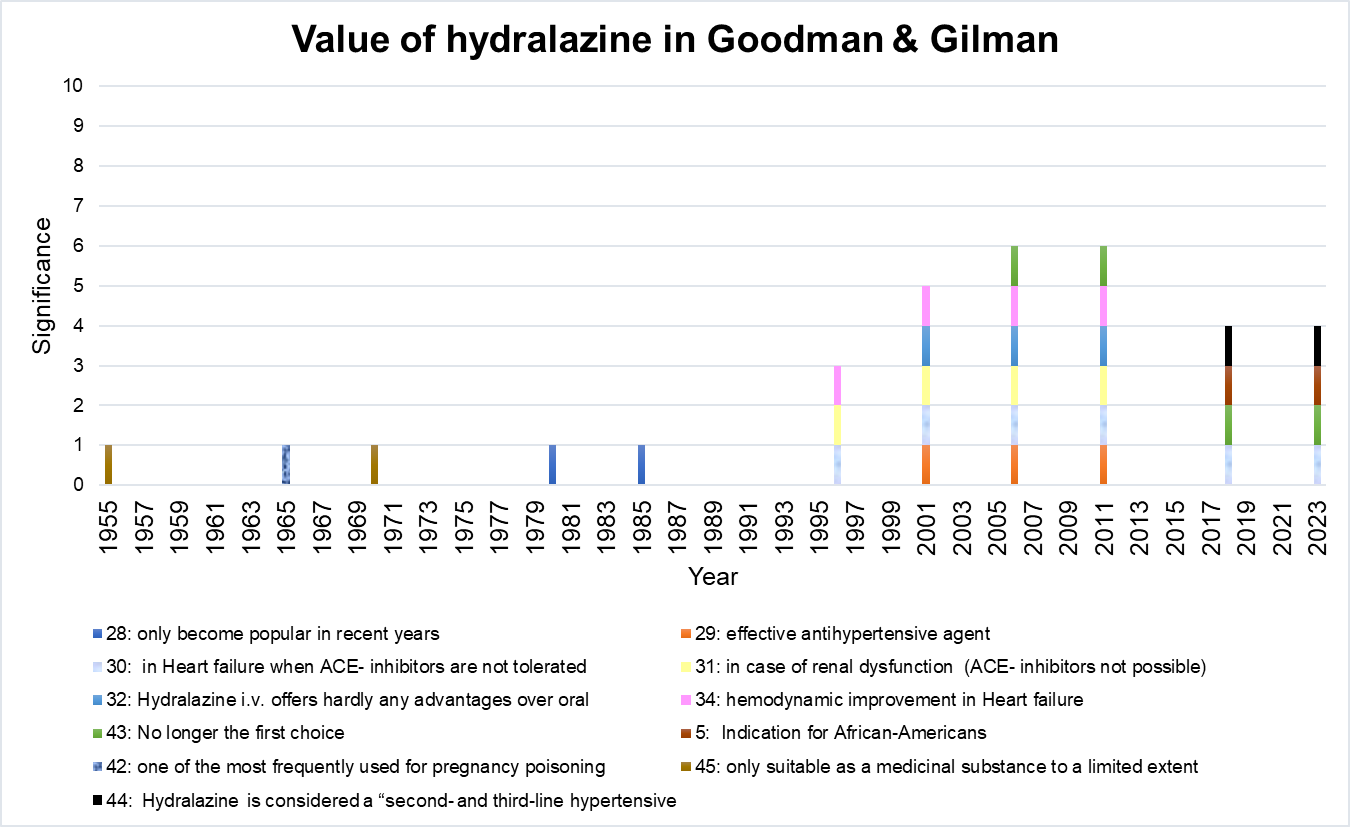
Figure S8:** Information on the change in place value in the textbook The Pharmacological Basis of Therapeutics (Goodman & Gilman)
